# Systemic inflammatory factors combined with HR-VWI-based plaque vulnerability discriminate ischemic phenotypes and predict prognosis in patients with intracranial atherosclerosis

**DOI:** 10.1186/s13244-026-02395-1

**Published:** 2026-09-05

**Authors:** Ke Lv, Huiying Wang, Ying Liu, Xunxiao Zhao, Shuxin Ma, Wencan Fu, Yue Cheng, Shuang Xia

**Affiliations:** 1 School of Medicine, Nankai University, Tianjin, China; 2 Department of Radiology, Medical Imaging Institute of Tianjin, Tianjin First Central Hospital, School of Medicine, Nankai University, Tianjin, China; 3 Department of General Practice II, Tianjin First Central Hospital, Tianjin, China; 4 Department of Radiology, First Central Hospital of Tianjin Medical University, Tianjin, China

**Keywords:** Acute ischemic stroke, Intracranial atherosclerotic stenosis, Delayed perfusion, Systemic inflammatory markers, High-resolution vessel wall imaging

## Abstract

**Background:**

The relationship between systemic inflammation, plaque vulnerability, and stroke phenotypes in intracranial atherosclerotic stenosis (ICAS) is unclear. This study investigated inflammatory markers’ association with high-risk plaques and their value in differentiating stroke phenotypes and predicting prognosis.

**Materials and methods:**

298 symptomatic ICAS patients were classified into acute ischemic stroke (AIS, *n* = 207) and delayed perfusion (DP, *n* = 91) groups using multimodal MRI. Plaque vulnerability was graded based on high-resolution vessel wall imaging, inflammatory indices calculated, and associations assessed using regression and mediation analysis.

**Results:**

AIS patients had higher systemic inflammation and more high-risk plaques (all *p* < 0.05). Each Systemic Inflammation Response Index (SIRI) unit increase was associated with 32.4% higher AIS risk (*p* = 0.027) and nearly threefold increased 90-day poor outcome risk (*p* < 0.001). In an exploratory mediation analysis aimed at understanding how these markers jointly differentiate the two phenotypes, the association between SIRI and AIS was partially (25.9%) statistically accounted for by high-risk plaque features. A comprehensive model integrating clinical, inflammatory, and imaging markers effectively differentiated AIS from DP (AUC = 0.794). In an exploratory prognostic analysis with a small number of poor outcomes, adding SIRI improved the AUC from 0.610 to 0.959, with internal validation supporting robustness (mean cross-validated AUC: 0.919–0.939), *p* < 0.05. Associations were more pronounced in males.

**Conclusions:**

Systemic inflammation is associated with ischemic phenotype in ICAS, and this association may be partially explained by plaque vulnerability. Combined assessment effectively differentiates stroke phenotypes and predicts prognosis, particularly in males, supporting multi-parametric models for early identification of at-risk patients.

**Key Points:**

- **Question**: Can systemic inflammatory markers combined with high-risk plaque features discriminate acute ischemic stroke from delayed perfusion and predict prognosis in patients with intracranial atherosclerotic stenosis?
- **Findings**: Systemic inflammation response index was independently associated with acute ischemic phenotypes and poor prognosis. Multi-parametric models improved phenotype discrimination (AUC = 0.794) and prognosis prediction (cross-validated AUC: 0.919–0.939).
- **Critical relevance statement**: Systemic inflammation response index is associated with acute ischemic stroke in intracranial atherosclerotic stenosis, an association partially mediated by high-risk plaque features. Combined assessment of inflammatory and imaging biomarkers improves stroke phenotyping and prognosis prediction, particularly in male patients.

**Graphical Abstract:**

**Figure:**
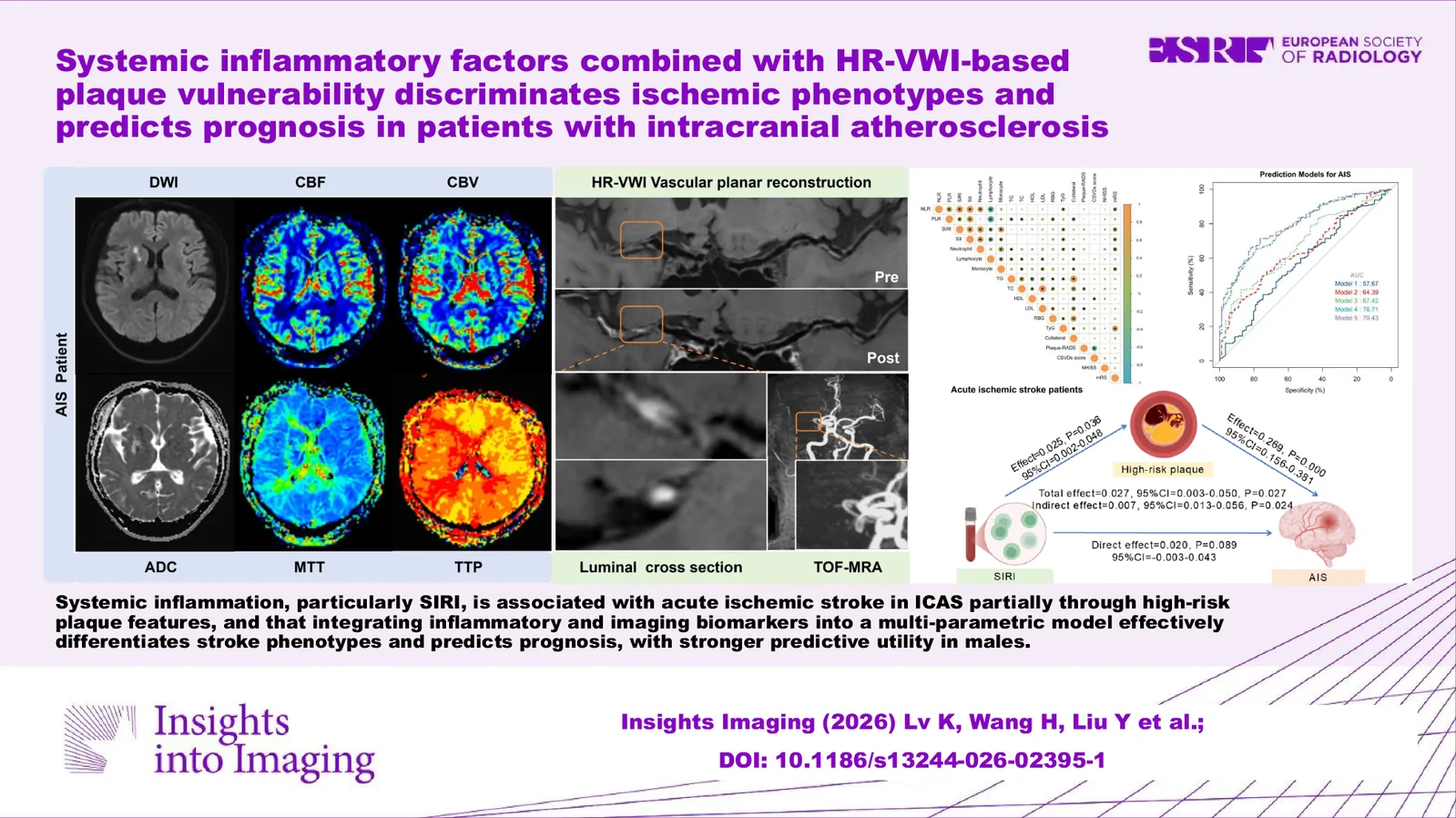

## Introduction

Acute ischemic stroke (AIS) is a leading global cause of disability and mortality, with complex and highly heterogeneous pathophysiology [1]. In Asian populations, intracranial atherosclerotic stenosis (ICAS) represents the most common etiology, making research into its pathogenesis crucial [2]. Systemic inflammation plays a central role in atherosclerosis, participating in plaque formation, instability, and rupture, which may lead to AIS [3–5].

Clinically accessible inflammatory indices—such as neutrophil-to-lymphocyte ratio (NLR), platelet-to-lymphocyte ratio (PLR), systemic inflammation response index (SIRI), and systemic immune-inflammation index (SII)—have demonstrated prognostic utility in cardiovascular and cerebrovascular diseases [5]. However, a systematic comparison of their predictive efficacy for different acute ischemic phenotypes within the same ICAS cohort is lacking. Concurrently, high-resolution vessel wall imaging (HR-VWI) enables detailed characterization of plaque morphology and high-risk features such as intraplaque hemorrhage and fibrous cap rupture [6, 7], with standardized grading systems for intracranial plaque vulnerability emerging [8].

In the acute setting, AIS presents with heterogeneous imaging phenotypes on multimodal MRI, reflecting distinct pathophysiological states [9]. Two key patterns are identifiable: (1) AIS, characterized by DWI hyperintensity; and (2) delayed perfusion (DP), defined by perfusion deficits without DWI lesions [10, 11]. DP patients exhibit hemodynamic impairment without established infarction, which may represent a potential therapeutic window for preventing progression to infarction, as patients with DWI‑negative/PWI‑positive findings have been shown to develop new infarction at short‑term follow‑up, although the natural history of DP remains incompletely understood [12]. It remains unknown whether systemic inflammatory profiles or their association with plaque vulnerability differ between AIS and DP patients.

We hypothesize that systemic inflammation, in conjunction with HR-VWI-defined high-risk plaque features, helps differentiate acute ischemic phenotypes and improves prognostic assessment. This retrospective study aims to: (1) compare inflammatory markers and culprit-plaque characteristics between AIS and DP patients; (2) evaluate the value of these markers, independently and synergistically, in differentiating AIS from DP, including exploration of gender-specific differences; and (3) investigate their prognostic value for predicting 90-day poor outcomes.

## Materials and methods

This retrospective study was approved by the Medical Research Ethics Committee of our hospital (No. 2019N155KY) and conducted in accordance with the Declaration of Helsinki. Written informed consent was obtained from all participants or their legal representatives.

### Participants

**Fig. 1:**
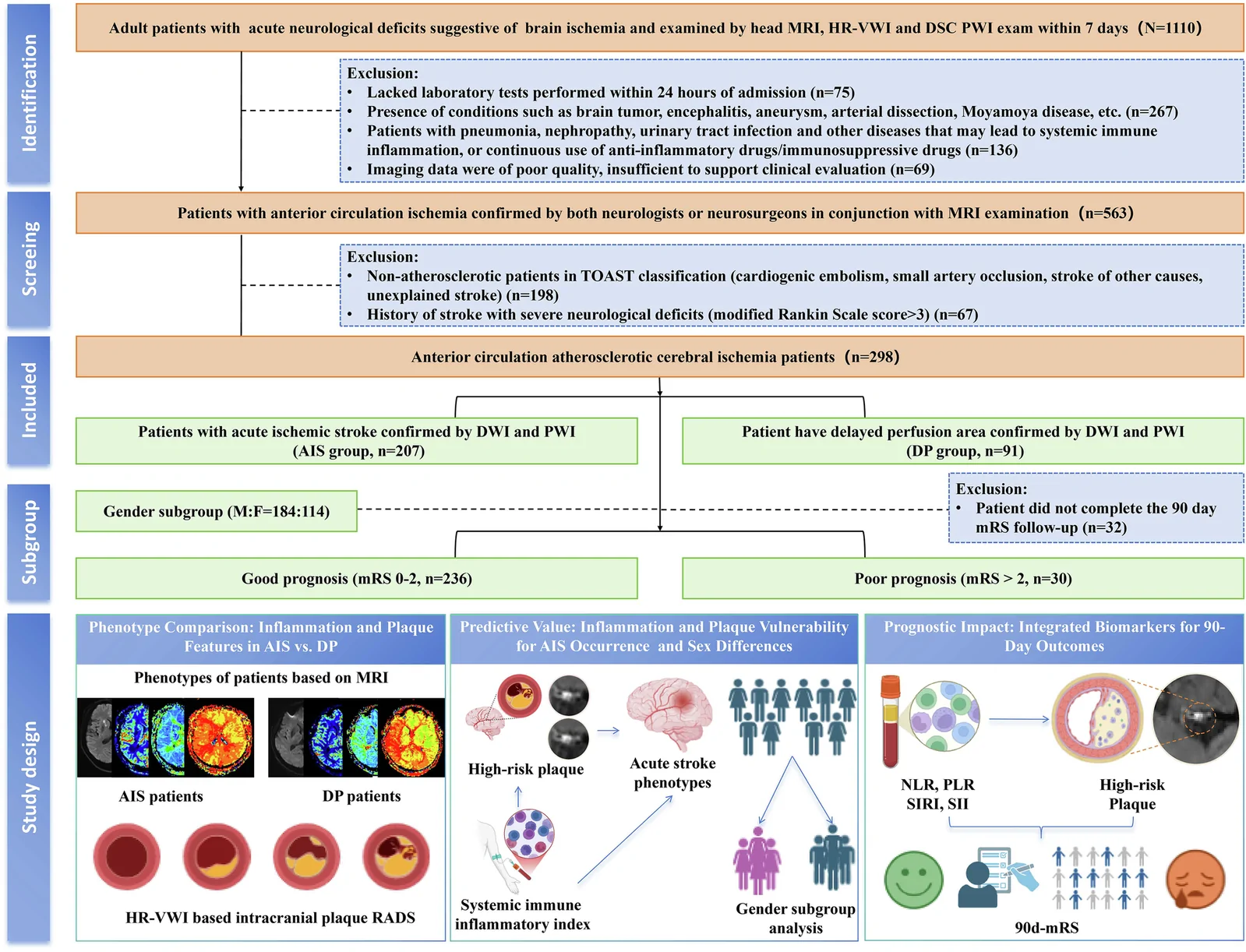
Flowchart of the study population and total study design

**Fig. 2:**
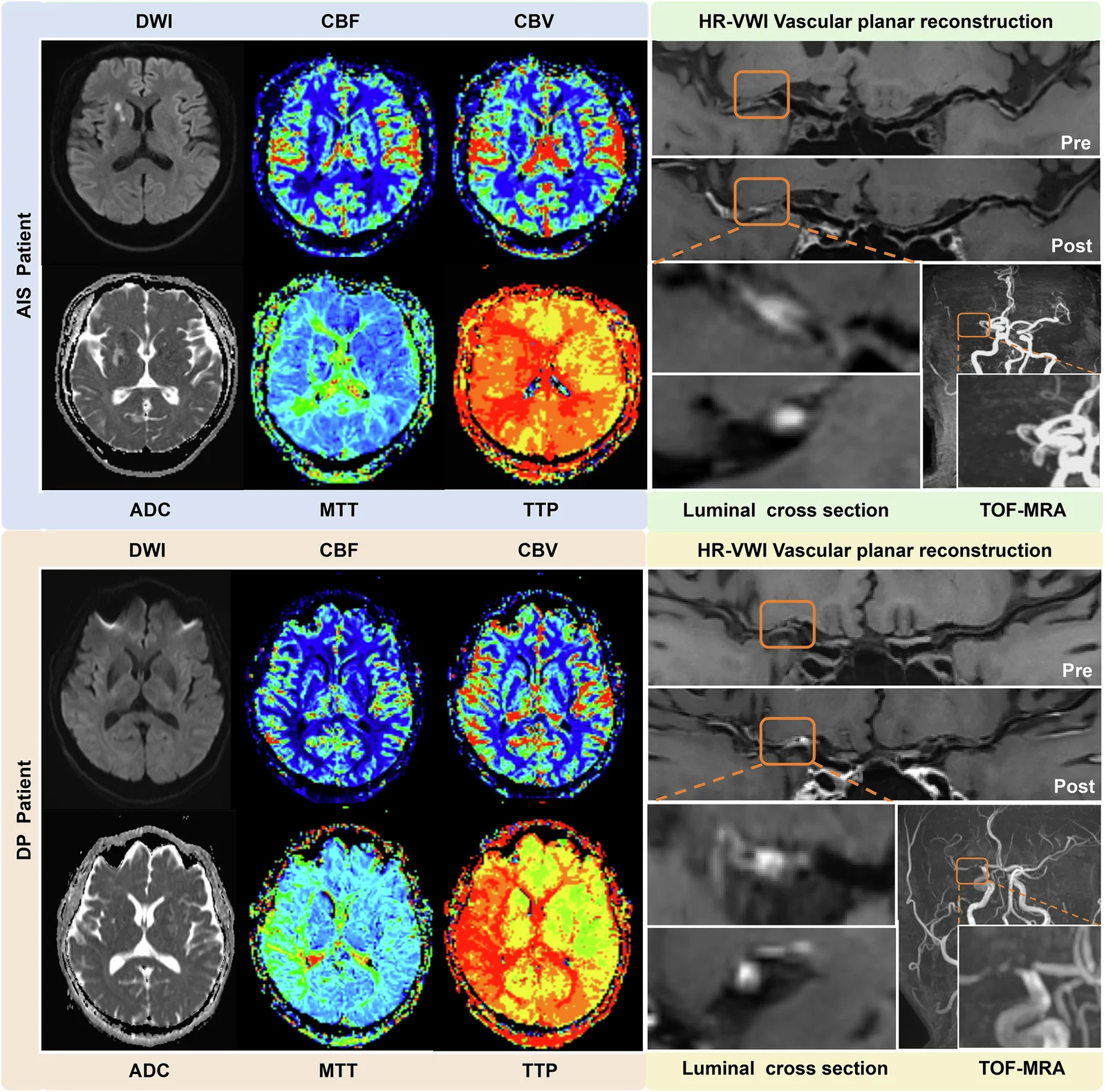
Example of patients’ images for acute ischemic stroke and delayed perfusion. AIS patients were identified by the presence of patchy hyperintensity on DWI with correspondingly reduced ADC values. Their PWI showed decreased CBF and CBV alongside prolonged MTT and TTP in the ischemic area. HR-VWI revealed a high-risk plaque in the right middle cerebral artery, characterized by pre-contrast intraplaque hyperintensity (suggesting hemorrhage) and marked post-contrast enhancement. This plaque caused local luminal occlusion and downstream segmental occlusion, warranting a Plaque-RADS 4 classification. DP patients were defined by the absence of DWI/ADC abnormalities. However, PWI demonstrated prolonged MTT and TTP in the symptomatic region. HR-VWI also identified a high-risk plaque in the same artery. It appeared irregular pre-contrast and showed marked enhancement post-contrast, resulting in severe local luminal stenosis while the downstream vessel remained patent. This plaque was also classified as Plaque-RADS 4. DWI, diffusion-weighted imaging; ADC, apparent diffusion coefficient; PWI, perfusion-weighted imaging; CBF, cerebral blood flow; CBV, cerebral blood volume; MTT, mean transit time; TTP, time to peak

Consecutive adult patients with acute neurological deficits suggestive of anterior circulation ischemia were retrospectively enrolled from our stroke center between 2020 and 2024. All patients underwent multimodal MRI (DWI, PWI, HR-VWI) within 7 days of symptom onset. Detailed inclusion/exclusion process is shown in Fig. 1. Group classification: AIS group—patients with DWI-positive lesions in the symptomatic vascular territory. DP group—patients with DWI-negative but PWI-positive findings (Tmax > 6 s), indicating hypoperfusion without established infarction. This group is clinically heterogeneous and may include patients with transient hypoperfusion, robust collateral compensation, post-ictal changes, or reversible ischemia without progression to infarction. Group assignment was performed independently by two neuroradiologists (6 and 10 years of experience), blinded to clinical data; disagreements were resolved by consensus with a third neuroradiologist (25 years of experience). Representative images are shown in Fig. 2.

### Clinical data

National Institutes of Health Stroke Scale (NIHSS) scores were assessed on admission by a senior neurologist (20 years of experience) and a neurology assistant (6 years), both blinded to imaging. The symptomatic side was jointly determined by the same physicians based on neurodiagnostic tests and medical histories, following American Heart Association/American Stroke Association criteria. Major risk factors for intracranial atherosclerotic stenosis (ICAS), including hypertension, hyperlipidemia, diabetes, coronary heart disease, atrial fibrillation, smoking, and drinking, were recorded [1].

### Follow-up assessment

Modified Rankin Scale (mRS) scores at 3 months were assessed by trained neurologists or research staff blinded to group assignment and baseline data, via structured telephone interviews or outpatient visits. mRS scores range from 0 (no symptoms) to 6 (death), with 0–2 defined as a favorable outcome and 3–6 as a poor outcome [4]. The specific steps for newly diagnosed DWI+ infarctions within 3 months in the DP group patients retrospectively supplemented can be found in the Supplementary Materials.

### Image protocol

MRI was conducted on a 3-T scanner (MAGNETOM Prisma, SIEMENS Healthcare) with a 64-channel head-and-neck coil. Head cushions and earplugs were used to ensure stability and reduce motion artifacts. The protocol included DWI, T2-FLAIR, TOF-MRA, DSC PWI, pre- and post-contrast HR-VWI. HR-VWI was acquired in the sagittal plane using IR-SPACE, with coverage from the whole brain to ≥ 2 cm below the carotid bifurcation. A gadolinium-based contrast agent (0.2 mL/kg, 4 mL/s) was administered intravenously. Post-contrast IR-SPACE was performed to assess plaque enhancement. Detailed scanning parameters are provided in Table S1.

### Image analysis—plaque identification and Plaque-RADS system

An atherosclerotic plaque was defined as focal wall thickening. The culprit plaque was identified as the only plaque present, or in cases with multiple plaques, the one causing the most severe stenosis within the symptomatic vascular territory. Two neuroradiologists (6 and 10 years of experience), blinded to clinical data, independently performed image analyses. Disagreements were resolved by consensus under the guidance of a senior neuroradiologist (25 years of experience).

**Fig. 3:**
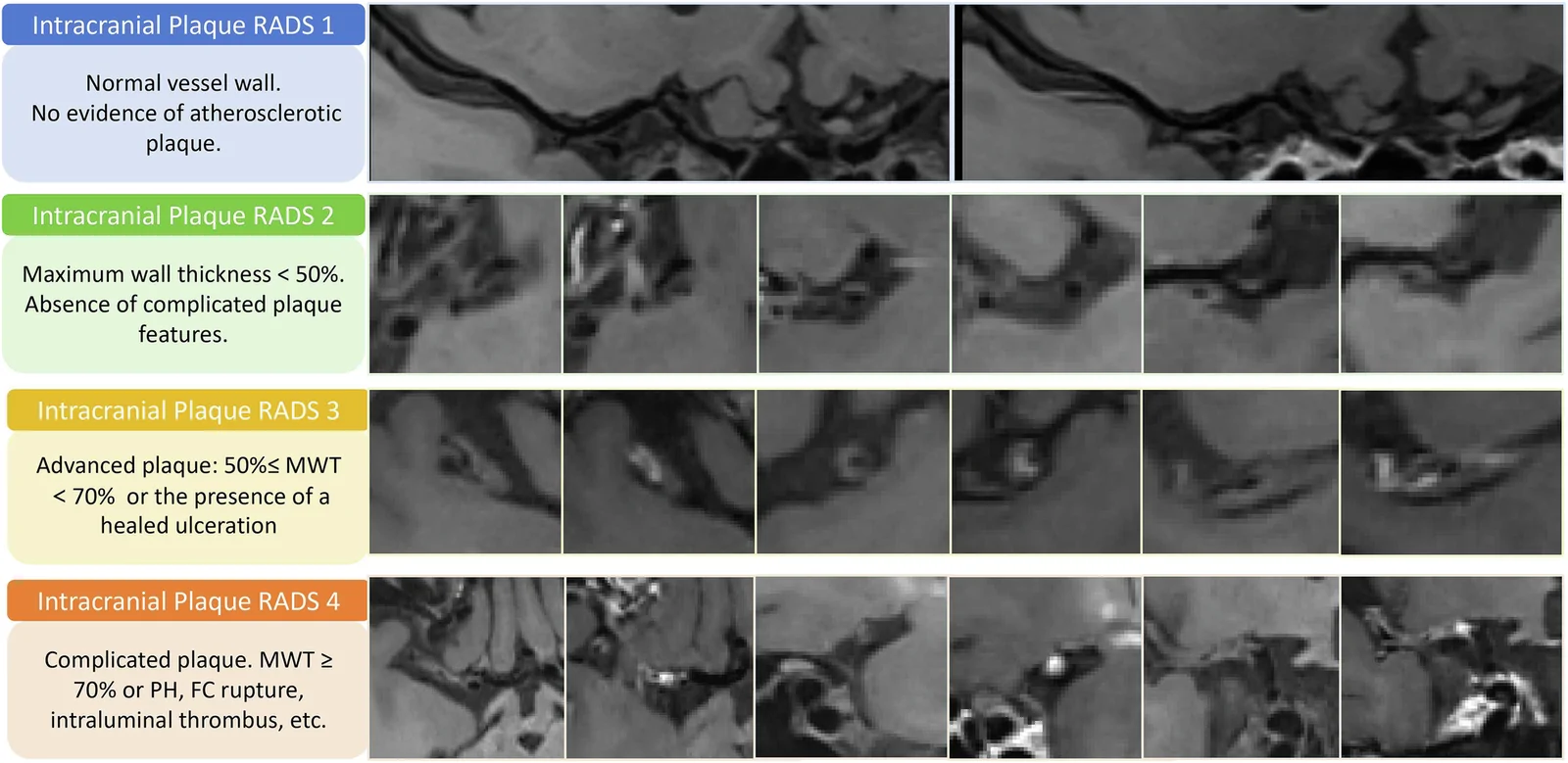
Grading of intracranial atherosclerotic plaque (using plaques in the M1 segment of the middle cerebral artery as examples). Intracranial Plaque-RADS 1 is defined as a normal vessel on HR-VWI without any positive plaque findings. Intracranial Plaque-RADS 2 is defined as a maximum wall thickness less than 50% of the luminal diameter, with no complex plaque features. Intracranial Plaque-RADS 3 is defined as a maximum wall thickness greater than 50% but less than 70% of the luminal diameter, with no complex plaque features. Intracranial Plaque-RADS 4 is defined as a maximum wall thickness greater than 70% of the luminal diameter, or the presence of any vulnerable plaque features, such as intraplaque hemorrhage (hyperintensity observed on pre-contrast T1 SPACE), ulceration (irregular plaque surface), or associated thrombus, etc

The evaluation of culprit plaques refers to the carotid Plaque-RADS grading criteria [7], and the following intracranial Plaque-RADS grading criteria have been developed. A summary of the grading criteria is provided below, while the representative patient examples are available in Fig. 3.

Plaque-RADS Grade 1: Normal vessel wall. No evidence of atherosclerotic plaque.

Plaque-RADS Grade 2: Maximum wall thickness (MWT) < 50% vessel diameter with absence of complicated plaque features.

Plaque-RADS Grade 3: Advanced plaque, defined by either: 50% ≤ MWT < 70% vessel diameter or the presence of a healed ulceration. Crucially, both types must lack features of a complicated plaque.

Plaque-RADS Grade 4: Complicated plaque, defined by either MWT ≥ 70% of the vessel diameter or the presence of any of the following high-risk features: intraplaque hemorrhage (IPH), fibrous cap (FC) rupture, or intraluminal thrombus.

Plaque-RADS grade 1–2 was defined as low-risk plaque, Plaque-RADS grade 3 was defined as middle-risk plaque, and Plaque-RADS grade 4 was defined as high-risk plaque. The scoring method for cerebral small vessel diseases (CSVDs) and the grading method for arterial collateral can be found in the Supplementary Materials [13, 14]. The evaluation of imaging indicators demonstrated good intra-observer and inter-observer agreement. Detailed results are presented in Table S2.

### Measurement of composite inflammatory ratios based on blood tests

Venous blood samples were collected within 24 h of admission. If multiple blood tests were performed during this period, the results from the first test were used. Data recorded included total cholesterol (TC), triglycerides (TG), high-density lipoprotein (HDL), low-density lipoprotein (LDL), random blood glucose (RBG), neutrophil (N), lymphocyte (L), platelet (PLT), and monocyte (M) counts. These values were used to derive the following inflammatory biomarkers and Triglyceride-glucose index (TyG) [7, 15].

Neutrophil-to-lymphocyte ratio (NLR = N/L)

Platelet-to-lymphocyte ratio (PLR = PLT/L)

Systemic inflammation response index (SIRI = N × [M/L])

Systemic immune-inflammation index (SII = PLT × [N/L])

Triglyceride-glucose index (TyG = Ln [Triglycerides × Blood glucose/2]).

### Statistical analysis

All analyses were performed using IBM SPSS Statistics (version 25.0) and R software (version 4.3.1). Continuous variables were compared using independent samples *t*-test or Mann–Whitney U test, and categorical variables using chi-square test. Correlations were assessed by Spearman’s rank correlation. Univariate logistic regression screened variables associated with outcomes; those with *p* < 0.05 were entered into multivariate logistic regression using backward stepwise selection, yielding adjusted odds ratios (OR) with 95% confidence intervals (CI). Mediation analysis was performed using the bootstrap method with 2000 resamples to examine whether high-risk plaque features mediated the relationship between systemic inflammation and acute ischemic stroke; given the cross-sectional design, results are hypothesis-generating. Discriminative ability of individual markers and combined models for predicting AIS and poor 90-day prognosis was evaluated by receiver operating characteristic (ROC) curves, with area under the curve (AUC) reported. DeLong’s test compared AUCs; net reclassification improvement (NRI) and integrated discrimination improvement (IDI) quantified incremental predictive value. To account for multiple comparisons, we applied the Benjamini–Hochberg false discovery rate (FDR) correction for exploratory analyses. For raw *p*-values reported as < 0.001, a conservative value of 0.001 was used for FDR calculation. A two-sided *p* < 0.05 was considered statistically significant.

## Results

### Baseline characteristics and differences between AIS and DP groups

**Fig. 4:**
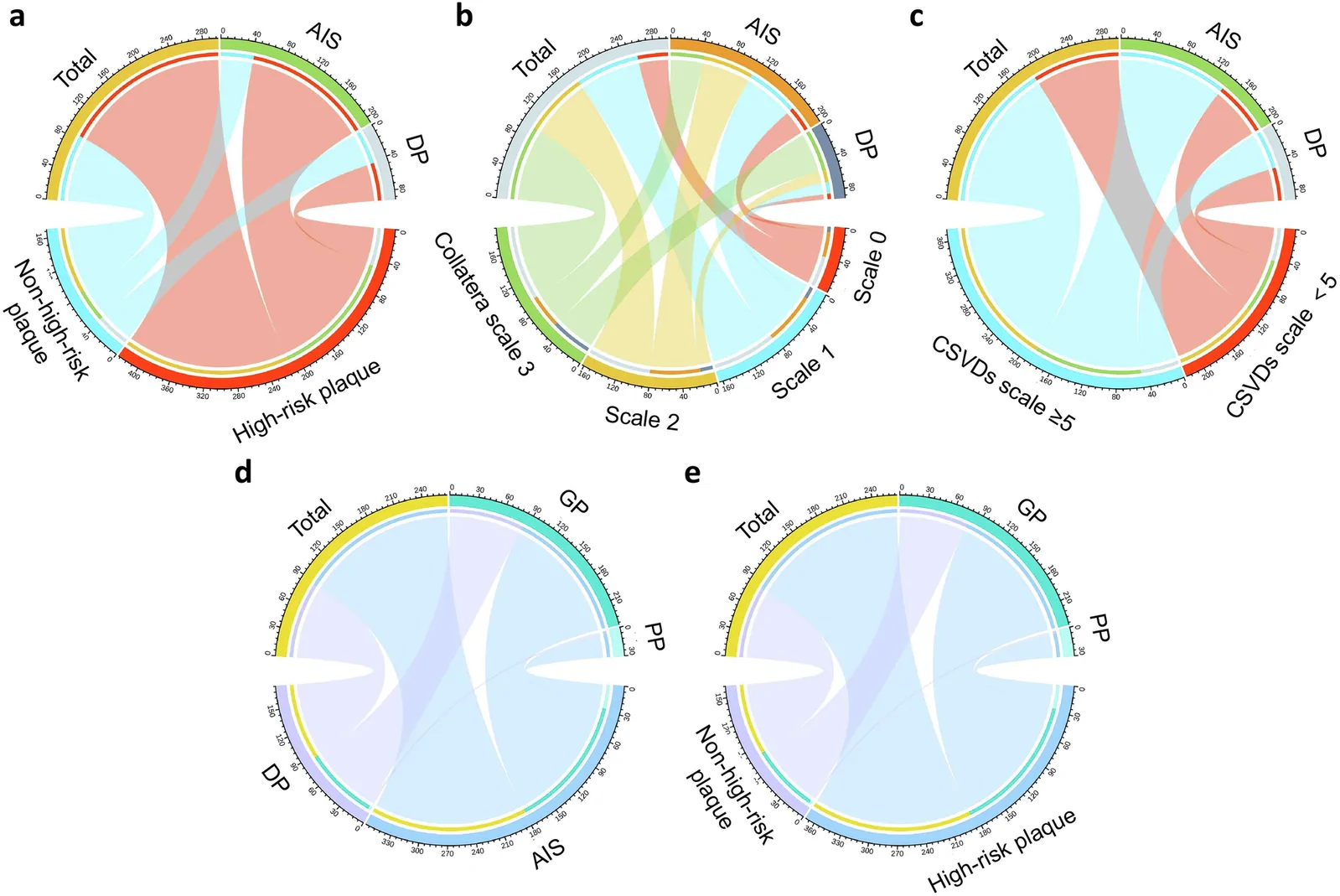
Chord diagrams depicting the distribution of imaging abnormalities among the overall patient cohort and different groups. **a** The distribution of high-risk culprit plaques in the acute ischemic stroke group and the delayed perfusion group. **b** The distribution of collateral grades in the acute ischemic stroke group and the delayed perfusion group. **c** The distribution of cerebral small vessel disease burden in the acute ischemic stroke group and the delayed perfusion group. **d** The distribution of AIS occurrence in different prognosis groups. **e** The distribution of high-risk culprit plaques in different prognosis groups. AIS, acute ischemic stroke; DP, delayed perfusion; CSVDs, cerebral small vessel diseases; GP, good prognosis; PP, poor prognosis

A total of 298 symptomatic patients with anterior circulation ICAS were included, with 207 in the AIS group and 91 in the DP group. The two groups were well-matched for age, sex, and traditional vascular risk factors (all *p*-adjust > 0.05). However, significant differences were observed in key biomarkers and imaging features. The AIS group showed elevated systemic inflammatory indices, including NLR, PLR, SIRI, and SII (all *p*-adjust < 0.05, Table 1 and Fig. S1). High-risk culprit plaques were substantially more prevalent in the AIS group (79.7% vs. 52.7%, *p*-adjust = 0.014), as was CSVDs burden (*p*-adjust = 0.034, Table 1 and Fig. 4a, c). Follow-up data revealed that among the DP patients, 38.5% (35/91) progressed to DWI+ new infarction within 3 months (see Supplementary Materials and Fig. S2).

**Table 1:** Clinical and imaging data of patients with different ischemic phenotypes

Variable	Total (*N* = 298)	Acute ischemic stroke group (*n* = 207)	Delayed-perfusion group (*n* = 91)	*p*-value	*p*-adjust
Age (years)	59.17 ± 12.48	59.26 ± 12.07	58.91 ± 13.43	0.813	0.999
Sex, female, *n* (%)	114 (38.3)	78 (37.7)	36 (39.6)	0.796	0.999
Vascular risk factors					
Hypertension, *n* (%)	232 (77.9)	162 (78.3)	70 (76.9)	0.880	0.999
Diabetes, *n* (%)	153 (51.3)	112 (54.1)	41 (45.1)	0.267	0.394
Hyperlipidemia, *n* (%)	91 (30.5)	66 (31.9)	25 (27.5)	0.496	0.661
Coronary heart disease, *n* (%)	71 (23.8)	50 (24.2)	21 (23.1)	0.884	0.969
Atrial fibrillation, *n* (%)	30 (10.1)	20 (9.7)	10 (11.0)	0.835	0.999
Smoking history, *n* (%)	169 (56.7)	120 (58.0)	49 (53.8)	0.528	0.672
Alcohol history, *n* (%)	139 (46.6)	102 (49.3)	37 (40.7)	0.207	0.322
NIHSS, median [IQR]	3 [1–4]	3 [2–5]	2 [1–4]	0.041*	0.088
mRS (0–2), *n* (%)	264 (88.6)	174 (84.1)	90 (97.9)	0.025*	0.058
Laboratory data					
TG, median [IQR]	1.35 [0.94–2.13]	1.36 [0.98–2.18]	1.33 [0.91–1.96]	0.118	0.219
TC, median [IQR]	3.99 [3.32–4.71]	4.06 [3.37–4.83]	3.78 [2.98–4.31]	0.011*	0.034^†^
HDL, median [IQR]	1.02 [0.85–1.26]	1.01 [0.87–1.25]	1.03 [0.81–1.29]	0.999	0.999
LDL, median [IQR]	2.40 [1.74–3.09]	2.50 [1.93–3.16]	2.11 [1.58–2.92]	0.010*	0.034^†^
RBG, median [IQR]	6.01 [5.00–7.53]	6.20 [5.02–8.23]	5.59 [4.97–6.54]	0.012*	0.034^†^
Neutrophil, median [IQR]	4.46 [3.32–5.69]	4.72 [3.48–5.81]	4.10 [3.16–5.12]	0.007*	0.034^†^
Lymphocyte, median [IQR]	1.65 [1.25–2.13]	1.61 [1.21–2.13]	1.71 [1.40–2.19]	0.125	0.219
Monocyte, median [IQR]	0.44 [0.34–0.56]	0.45 [0.36–0.57]	0.42 [0.32–0.51]	0.139	0.322
Platelet, median [IQR]	230.50 [187.50–276.25]	231.00 [188.00–285.00]	230.00 [186.00–272.00]	0.356	0.498
TyG index	1.44 [1.06–1.88]	1.49 [1.09–1.96]	1.35 [0.88–1.71]	0.014*	0.036^†^
Inflammatory index					
NLR, median [IQR]	2.57 [1.86–3.90]	2.78 [2.05–4.25]	2.19 [1.61–3.43]	0.002*	0.019^†^
PLR, median [IQR]	136.76 [108.91–190.06]	143.79 [111.97–210.91]	121.24 [98.38–158.99]	0.010*	0.034^†^
SIRI, median [IQR]	1.18 [0.78–1.87]	1.29 [0.82–2.01]	1.04 [0.65–1.55]	0.010*	0.034^†^
SII, median [IQR]	591.80 [414.16–958.05]	633.73 [430.31–986.28]	478.55 [343.44–817.28]	0.001*	0.014^†^
Plaque-RADS					
High-risk plaque, *n* (%)	213 (71.5)	165 (79.7)	48 (52.7)	< 0.001**	0.014^†^
Collateral scale, *n* (%)				0.056	0.112
0	39 (13.1)	32 (15.5)	7 (7.7)		
1	82 (27.5)	67 (32.4)	15 (16.5)		
2	81 (27.2)	65 (31.4)	16 (17.6)		
3	96 (33.2)	43 (20.8)	53 (58.2)		
CSVDs score, median [IQR]	6 [2–7]	6 [3–7]	5 [1–6]	0.008*	0.034^†^

*NIHSS* National Institutes of Health Stroke Scale, *mRS* modified Rankin Scale, *TG* triglycerides, *TC* total cholesterol, *LDL* low-density lipoprotein cholesterol, *HDL* high-density lipoprotein cholesterol, *RBG* random blood glucose, *NLR* neutrophil-to-lymphocyte ratio, *PLR* platelet-to-lymphocyte ratio, *SIRI* systemic inflammation response index, *SII* systemic immune-inflammation index, *TyG* triglyceride glucose index, *CSVDs* cerebral small vessel diseases

* *p* < 0.05

** *p* < 0.001

^†^ FDR-adjusted *p*-adjust < 0.05 (Benjamini–Hochberg correction)

Of 298 initially enrolled patients, 266 (89.3%) completed the 90-day follow-up. 236 had a good prognosis (mRS 0–2) and 30 had a poor prognosis (mRS > 2). The poor prognosis group had significantly higher NIHSS, neutrophil count, monocyte count, NLR, PLR, SIRI, and SII (all *p*-adjust < 0.05, Table S3), as well as higher proportions of DWI-confirmed AIS (*p*-adjust = 0.025) and high-risk plaques (*p*-adjust = 0.025, Table S4 and Fig. 4d, e).

### Correlations between inflammatory markers, clinical severity, and imaging features

**Fig. 5:**
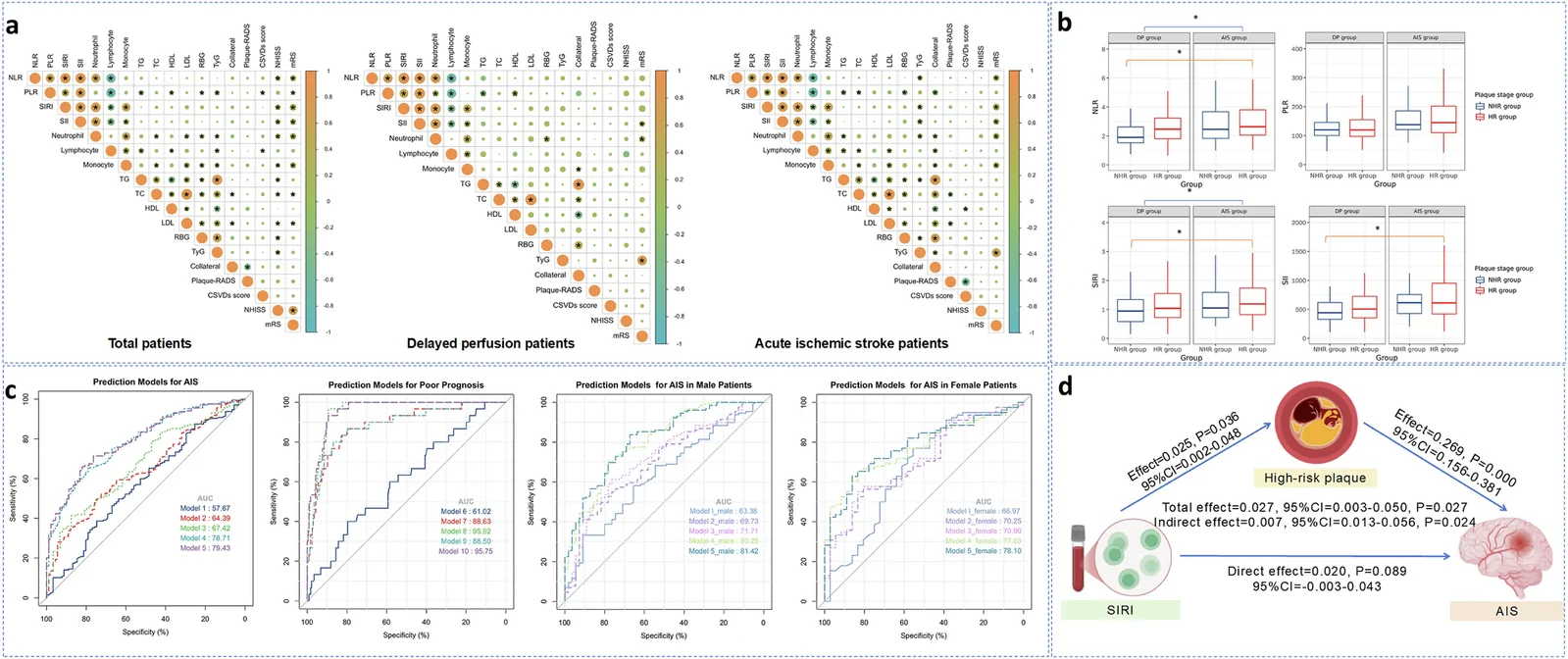
Patient data correlation, subgroup comparison, receiver operating characteristic (ROC) analysis, and mediation effect analysis results. **a** The correlation between clinical data, laboratory tests, and imaging parameters of all patients, AIS group, and DP group. **b** The subgroup distribution of inflammatory factors (NLR, PLR, SIRI, SII) and the comparison results between groups. **c** ROC curves of acute ischemic stroke occurrence, poor prognosis, and sex heterogeneity acute ischemic stroke occurrence. **d** The mediation analysis results, showing that the association between SIRI and AIS was partially explained by high-risk plaque features. NLR, neutrophil-to-lymphocyte ratio; PLR, platelet-to-lymphocyte ratio; RBG, random blood glucose; SIRI, systemic inflammation response index; SII, systemic immune-inflammation index; NHR group, non-high-risk plaque group; HR group, high-risk plaque group. * *p* < 0.05; ** *p* < 0.001

Correlation analyses revealed distinct relationships between inflammatory markers and disease characteristics across cohorts. After FDR adjustment, in the overall cohort, SIRI was positively correlated with NIHSS score (r = 0.239, *p*-adjust = 0.001) and 90-day mRS score (r = 0.352, *p*-adjust = 0.001). SII was also positively correlated with NIHSS score (r = 0.207, *p*-adjust = 0.001) and 90-day mRS score (r = 0.280, *p*-adjust = 0.001). PLR was positively correlated with CSVDs score (r = 0.136, *p*-adjust = 0.032). In the DP subgroup, SIRI was positively correlated with 90-day mRS score (r = 0.310, *p*-adjust = 0.002). In the AIS subgroup, NLR (r = 0.291, *p*-adjust = 0.002), SIRI (r = 0.355, *p*-adjust = 0.002), and SII (r = 0.270, *p*-adjust = 0.002) were all positively correlated with 90-day mRS score (Fig. 5a).

### Independent associations of inflammatory markers with AIS and prognosis

In univariable analysis, several inflammatory markers showed associations with AIS. However, after FDR correction for multiple comparisons, only NIHSS, several laboratory parameters (TC, LDL, neutrophil count, RBG, TyG index), and imaging markers (high-risk plaque, collateral scale, CSVDs score) remained statistically significant. SIRI showed a nominal association with AIS in univariable analysis (OR = 1.309, 95% CI 1.026–1.669, nominal *p* = 0.030; *p*-adjust = 0.054). In multivariable analysis adjusting for confounders, SIRI (adjusted OR = 1.324, 95% CI 1.033–1.696, *p* = 0.027) remained independently associated with AIS (Table 2). In the analysis of poor prognosis, NLR, PLR, SIRI, and SII were independently associated after comprehensive adjustment, with SIRI showing the strongest association (adjusted OR = 2.728, 95% CI 1.888–3.941, *p* < 0.001) (Table 3).

**Table 2:** Univariate and multivariate logistic analysis for acute ischemic stroke occurrence

Variable	Univariable analysis				Multivariable analysis		
	OR value	95% CI	*p*-value	*p*-adjust	OR value	95% CI	*p*-value
NIHSS	1.366	1.179–1.582	< 0.001**	0.018^†^	1.390	1.185–1.630	< 0.001**
Laboratory data							
TG	1.248	0.960–1.621	0.098	0.136	1.235	0.939–1.624	0.131
TC	1.362	1.094–1.696	0.006*	0.018^†^	1.368	1.083–1.727	0.008*
HDL	0.822	0.433–1.561	0.549	0.581	0.838	0.434–1.617	0.598
LDL	1.493	1.138–1.958	0.004**	0.018^†^	1.478	1.111–1.965	0.007*
RBG	1.135	1.018–1.265	0.023*	0.046^†^	1.131	1.000–1.280	0.051
Neutrophil	1.227	1.066–1.413	0.004*	0.018^†^	1.238	1.070–1.432	0.004*
Lymphocyte	0.776	0.546–1.103	0.157	0.202	0.763	0.526–1.106	0.153
Monocyte	2.621	0.656–10.478	0.173	0.208	2.912	0.709–11.962	0.138
Platelet	1.001	0.998–1.004	0.658	0.658	1.001	0.998–1.004	0.437
TyG index	1.702	1.138–2.544	0.010*	0.023^†^	1.720	1.095–2.702	0.019*
Inflammatory index							
NLR	1.118	0.993–1.258	0.065	0.098	1.115	0.988–1.259	0.077
PLR	1.002	0.999–1.005	0.292	0.329	1.002	0.999–1.005	0.229
SIRI	1.309	1.026–1.669	0.030*	0.054	1.324	1.033–1.696	0.027*
SII	1.000	1.000–1.001	0.057	0.093	1.000	1.000–1.001	0.049*
Plaque-RADS							
High-risk plaque	3.519	2.065–5.997	< 0.001**	0.018^†^	3.846	2.214–6.681	< 0.001**
Collateral scale	0.498	0.381–0.653	< 0.001**	0.018^†^	0.473	0.357–0.625	< 0.001**
CSVDs score	1.119	1.032–1.214	0.006*	0.018^†^	1.160	1.052–1.278	0.003*

The multivariate logistic regression model has been adjusted for sex, age, and vascular risk factors (hypertension, diabetes, hyperlipidemia, coronary heart disease, atrial fibrillation, smoking history, and drinking history)

*NIHSS* National Institutes of Health Stroke Scale, *TG* triglycerides, *TC* total cholesterol, *LDL* low-density lipoprotein cholesterol, *HDL* high-density lipoprotein cholesterol, *RBG* random blood glucose, *NLR* neutrophil-to-lymphocyte ratio, *PLR* platelet-to-lymphocyte ratio, *SIRI* systemic inflammation response index, *SII* systemic immune-inflammation index, *TyG* triglyceride glucose index, *CSVDs* cerebral small vessel diseases

* *p* < 0.05

** *p* < 0.001

^†^ FDR-adjusted *p*-adjust < 0.05 (Benjamini–Hochberg correction)

**Table 3:** Univariate and multivariate logistic analysis for clinical prognosis

Variable	Univariable analysis				Multivariable analysis		
	OR value	95% CI	*p*-value	*p*-adjust	OR value	95% CI	*p*-value
NIHSS	1.844	1.508–2.256	< 0.001**	0.019^†^	1.991	1.584–2.502	< 0.001**
Laboratory data							
TG	1.004	0.692–1.457	0.983	1.000	1.009	0.670–1.521	0.956
TC	1.029	0.749–1.413	0.860	0.961	0.976	0.689–1.382	0.891
HDL	2.174	0.990–4.776	0.053	0.093	2.445	1.069–5.590	0.034*
LDL	1.109	0.763–1.613	0.586	0.742	1.028	0.688–1.540	0.889
RBG	1.088	0.856–1.237	0.201	0.294	1.092	0.942–1.267	0.245
Neutrophil	1.818	1.464–2.259	< 0.001**	0.019^†^	1.920	1.517–2.431	< 0.001**
Lymphocyte	0.637	0.345–1.174	0.148	0.234	0.666	0.352–1.258	0.210
Monocyte	5.260	1.950–14.210	< 0.001**	0.019^†^	5.810	2.090–16.160	< 0.001**
Platelet	1.004	1.000–1.007	0.049*	0.093	1.004	1.000–1.008	0.028*
TyG index	1.012	0.547–1.874	0.970	1.000	0.902	0.434–1.878	0.784
Inflammatory index							
NLR	1.375	1.169–1.616	< 0.001**	0.019^†^	1.390	1.182–1.633	< 0.001**
PLR	1.005	1.001–1.009	0.006*	0.019^†^	1.006	1.002–1.010	0.004*
SIRI	2.643	1.869–3.738	< 0.001**	0.019^†^	2.728	1.888–3.941	< 0.001**
SII	1.001	1.001–1.002	< 0.001**	0.019^†^	1.002	1.001–1.002	< 0.001**
Acute ischemic stroke	4.974	1.465–16.884	0.010*	0.024^†^	5.136	1.493–17.668	0.009*
High-risk plaque	4.443	1.307–15.098	0.017**	0.036^†^	5.136	1.493–17.668	0.009*
Collateral scale	0.842	0.593–1.195	0.336	0.456	4.389	1.274–15.120	0.019*
CSVDs score	1.032	0.914–1.165	0.613	0.742	0.990	0.859–1.142	0.892

The multivariate logistic regression model has been adjusted for sex, age, and vascular risk factors (hypertension, diabetes, hyperlipidemia, coronary heart disease, atrial fibrillation, smoking history, and drinking history)

*NIHSS* National Institutes of Health Stroke Scale, *TG* triglycerides, *TC* total cholesterol, *LDL* low-density lipoprotein cholesterol, *NLR* neutrophil-to-lymphocyte ratio, *PLR* platelet-to-lymphocyte ratio, *SIRI* systemic inflammation response index, *SII* systemic immune-inflammation index, *TyG* triglyceride glucose index, *CSVDs* cerebral small vessel diseases

* *p* < 0.05

** *p* < 0.001

^†^ FDR-adjusted *p*-adjust < 0.05 (Benjamini–Hochberg correction)

### Synergistic interaction between systemic inflammation and plaque vulnerability

The risk associated with inflammation was modulated by local plaque phenotype. Patients with high-risk plaques had significantly higher NLR and SIRI levels than those with low-risk plaques (both *p*-adjust < 0.05, Table S5). Notably, the highest inflammatory burden was observed in AIS patients with high-risk culprit plaques, who showed significantly elevated RBD, neutrophil count, NLR, SIRI, and SII compared to DP patients with low-risk plaques (all *p* < 0.05, Table S6 and Figs. 5b, S3), suggesting a synergistic interaction between systemic inflammation and local plaque instability in precipitating infarction.

To explore whether inflammatory and imaging markers could help differentiate between AIS and DP, mediation analysis was performed to examine the interrelationships among these markers. The results showed that the distinction of AIS from DP mediated by SIRI can be explained by advanced plaque features. SIRI had a significant total effect on AIS phenotype (c = 0.027, *p* = 0.027), comprising a non-significant direct effect (c’ = 0.020, *p* = 0.089) and a significant indirect effect through advanced plaques (a × b = 0.007, *p* = 0.024), accounting for 25.9% of the total effect. These findings suggest that the ability of elevated SIRI to differentiate AIS from DP is fully explained by its association with high-risk plaque characteristics (Fig. 5d). No significant mediation effects were observed for NLR, PLR, or SII (Fig. S4a–c). A reverse model, testing whether plaque features distinguish the phenotypes through SIRI, was not supported (although the indirect effect was significant, a × b = 0.012, *p* = 0.023, the path from SIRI to AIS failed to reach significance, b = 0.020, *p* = 0.089), confirming that the discriminatory capacity is specific to the pathway from inflammation to plaque features, not the reverse (Fig. S4d).

### Incremental predictive value of a multi-parametric model

To evaluate clinical utility, sequential prediction models were constructed. Adding inflammatory and metabolic markers (TyG index, SIRI, SII) to a baseline clinical model significantly improved discrimination for AIS occurrence (Model 3 AUC: 0.674 vs. Model 2 AUC: 0.635; DeLong’s test *p* < 0.05) (Tables 4, S7, S8). The fully integrated model (Model 5), incorporating clinical, inflammatory, metabolic, and imaging parameters (high-risk plaque, collateral status, CSVDs burden), achieved the highest predictive performance for AIS (AUC = 0.794, 95% CI 0.742–0.846), with NRI = 86.5% and IDI = 0.210 (Tables 4, S8 and Fig. 5c).

**Table 4:** Risk factors and predictive models for acute ischemic stroke occurrence and poor prognosis

Variable	Area	Standard error	*p*-value	95% CI		Hosmer–Lemeshow test	
				Lower bound	Upper bound	Chi-square	*p*-value
Differentiation of stroke phenotypes (acute ischemic stroke vs. delayed perfusion)							
Model 1	0.564	0.036	0.079	0.493	0.636	7.296	0.505
Model 2	0.635	0.034	< 0.001	0.569	0.701	9.781	0.281
Model 3	0.674	0.033	< 0.001	0.610	0.738	8.493	0.387
Model 4	0.787	0.027	< 0.001	0.734	0.840	1.030	0.998
Model 5	0.794	0.027	< 0.001	0.742	0.846	2.349	0.968
Predictive models for poor prognosis							
Model 6	0.610	0.054	0.049	0.505	0.716	6.489	0.593
Model 7	0.886	0.034	< 0.001	0.820	0.952	5.388	0.715
Model 8	0.959	0.012	< 0.001	0.936	0.982	4.812	0.778
Model 9	0.885	0.035	< 0.001	0.817	0.953	4.851	0.773
Model 10	0.957	0.012	< 0.000	0.923	0.982	3.005	0.934
Differentiation of stroke phenotypes in male patients							
Model l_male	0.634	0.043	0.004	0.549	0.719	4.548	0.805
Model 2_male	0.697	0.041	< 0.001	0.616	0.778	7.586	0.475
Model 3_male	0.757	0.030	< 0.001	0.678	0.826	7.632	0.470
Model 4_male	0.803	0.035	< 0.001	0.734	0.872	9.606	0.294
Model 5_male	0.814	0.034	< 0.001	0.748	0.880	3.770	0.877
Differentiation of stroke phenotypes in female patients							
Model l_female	0.670	0.058	0.004	0.557	0.783	5.685	0.682
Model 2_female	0.702	0.050	0.001	0.604	0.801	6.453	0.597
Model 3_female	0.709	0.050	< 0.001	0.611	0.807	18.251	0.019
Model 4_female	0.770	0.043	< 0.001	0.685	0.855	11.831	0.159
Model 5_female	0.781	0.043	< 0.001	0.696	0.866	2.744	0.949

For Outcome 1 (Differentiation of stroke subtypes): Model 1 adjusted for demographic characteristics and vascular risk factors; Model 2 added the NIHSS score to Model 1; Model 3 further added metabolic/inflammatory markers (TyG index, SIRI, SII) to Model 2; Model 4 added imaging markers (high-risk plaque, collateral scale, CSVDs score) to Model 2; and Model 5 added the aforementioned imaging markers to Model 3

For Outcome 2 (Predicting poor prognosis): Model 6 adjusted for demographic characteristics and vascular risk factors; Model 7 further adjusted NIHSS based on Model 6; Model 8 added SIRI to Model 7; Model 9 added high-risk plaque to Model 7; Model 10 added high-risk plaque to Model 8. Prognostic models for poor prognosis (Models 6–10) were based on only 30 patients with poor outcomes. The high AUC values should be interpreted with caution due to the risk of overfitting

Male subgroup (Differentiation of stroke subtypes): Sequential models were built starting from a base model (demographics and vascular risk factors), with the NIHSS score added next, followed by inflammatory markers (NLR, SIRI, SII), and finally imaging markers (high-risk plaque, collateral scale, CSVDs score), culminating in a full model containing all variables

Female subgroup (Differentiation of stroke subtypes): Using the same base model structure, sequential adjustments were made by adding the NIHSS score, followed by metabolic/inflammatory markers (NLR, PLR, SIRI, SII), and then imaging markers (high-risk plaque, collateral scale), resulting in a final comprehensive model

*SIRI* systemic inflammation response index, *SII* systemic immune-inflammation index, *NLR* neutrophil-to-lymphocyte ratio, *PLR* platelet-to-lymphocyte ratio, *TyG* triglyceride-glucose index, *CSVDs* cerebral small vessel diseases, *NIHSS* National Institutes of Health Stroke Scale

Due to the small number of patients in the poor prognosis group, prognostic analyses are presented as exploratory. Only SIRI and high-risk plaque—which had higher odds ratios in multivariable analysis—were selected for the poor prognosis prediction model. Adding SIRI (Model 8) significantly improved predictive efficacy over the clinical epidemiological model (Model 6) (AUC = 0.959, 95% CI 0.936–0.982), representing an AUC increase of 0.349 (DeLong’s test *p* < 0.001), with NRI = 154.58% and IDI = 0.488 (Tables 4, S7, S8 and Fig. 5c). Internal validation results (detailed in the Supplementary Materials) consistently demonstrated robust model performance, with mean cross-validated AUCs ranging from 0.919 to 0.939 and optimism of 2.1–4.2%. However, given the limited number of poor outcomes, these AUC estimates are likely optimistic and should be interpreted as hypothesis-generating, pending external validation.

### Sex heterogeneity in the predictive utility of inflammatory markers

Stratified analyses revealed a marked sex disparity. Male patients had significantly higher SIRI levels than females (*p* = 0.017, Tables S9, S10 and Fig. S5). In multivariable analyses stratified by sex, elevated NLR, SIRI, and SII were independently associated with AIS occurrence in males (all *p* < 0.05), but not in females (Table S11). Consequently, incorporating inflammatory markers significantly improved model discrimination only in males (AUC increase from 0.634 to 0.757, NRI = 43.6%, IDI = 0.074; *p* < 0.05) (Fig. 5c and Tables 4, S8), with no significant improvement in females. These results demonstrate a fundamental sex‑specific difference in the pathophysiological contribution of systemic inflammation to AIS risk.

## Discussion

This retrospective cohort study investigated differences in systemic inflammation and intracranial atherosclerotic plaque characteristics between two acute cerebral ischemic phenotypes (AIS vs. DP), as well as their value for predicting prognosis. The principal findings are: (1) Systemic inflammatory indices (NLR, SIRI, SII) and the TyG index were significantly elevated in AIS patients compared to those with DP. (2) Systemic inflammation, particularly SIRI, in conjunction with high-risk plaque features, helped differentiate AIS from DP. Exploratory mediation analysis further suggested that the discriminatory effect of SIRI was partially (25.9%) statistically accounted for by high-risk plaque features, though this finding is hypothesis-generating given the cross-sectional design. (3) Comprehensive models integrating inflammatory, metabolic, and imaging markers demonstrated strong performance in differentiating AIS from DP and showed prognostic value for predicting 90-day poor outcomes. (4) Significant sex heterogeneity was observed, with inflammatory markers showing stronger differentiation ability in male patients.

Elevated NLR, SIRI, and SII in the AIS group highlight the central role of neutrophilia and relative lymphopenia in acute cerebral infarction, reflecting a systemic pro-inflammatory and potentially immunosuppressive state [16]. Ma et al reported that SIRI and SII are independent predictors of poor 90-day prognosis in AIS patients undergoing intravenous thrombolysis, correlating with stroke severity [7]. McCabe et al confirmed the significance of inflammatory markers for stroke recurrence in a meta-analysis [17]. Basic studies have shown that during acute AIS, blood-brain barrier disruption and neutrophil aggregation in ischemic brain tissue release inflammatory factors, damaging endothelial and basement membranes and exacerbating infarct core formation [18, 19]. This systemic inflammation likely promotes local neuroinflammation and blood-brain barrier dysfunction, exacerbating CNS injury. Clinically, there is an urgent need for convenient, stable inflammatory markers to elucidate mechanisms affecting infarct formation and prognosis. Indices like SII and SIRI, which integrate multiple leukocyte lineages, may more comprehensively capture systemic immune dysregulation following significant ischemia. Their strong correlation with NIHSS and mRS scores supports their role as biomarkers of ischemic burden and prognosis. The association of the TyG index with AIS aligns with insulin resistance as a key metabolic driver of inflammation and endothelial dysfunction, contributing to a vulnerable vascular milieu [14].

This study identified high-risk plaque features as a strong independent predictor of AIS. Moreover, a notable association between inflammatory status and plaque vulnerability was observed: among AIS patients, those with high-risk plaques had the highest levels of inflammatory markers. Mediation analysis further suggested that the relationship between systemic inflammation (particularly SIRI) and AIS was partially attributable to high-risk plaque features. This finding aligns with the hypothesized “dual-hit” mechanism, wherein a systemic pro-inflammatory state may destabilize locally vulnerable plaques, while inflammatory mediators from plaque rupture could exacerbate systemic inflammation, potentially creating a feedback loop [20, 21]. Such a cycle may underpin the progression from hemodynamic compromise to irreversible infarction, though prospective studies are needed to confirm this dynamic. The interplay between local and systemic inflammation is a growing focus in neuroimmunology. As emphasized by Shi and Yong, T cell-microglia/macrophage interactions drive neuroinflammation across neurological disorders [22]. Our findings provide clinical-imaging evidence aligning with this concept, suggesting that systemic inflammatory markers and imaging-defined plaque vulnerability are collectively associated with AIS risk.

Although individual indicators (SIRI, high-risk plaque features) have predictive value, the integrated model combining clinical factors, inflammatory/metabolic markers, and imaging burden achieved the highest predictive performance (with significant improvements in AUC, NRI, and IDI) in the analysis of AIS occurrence. For prognostic prediction of poor 90-day outcomes, the sample size was limited; the very high AUC values (e.g., 0.959) should be interpreted with caution due to potential overfitting. Internal validation confirmed robustness (mean cross-validated AUC: 0.919–0.939), though these findings remain exploratory pending external validation. Traditional risk models often overlook intracranial plaque characteristics and systemic inflammation [23]. Our model suggests that in patients with hemodynamic impairment (DP), combining HR-VWI to assess plaque burden with systemic inflammatory markers may help identify those at higher risk of AIS. Hemodynamic impairment without infarction may represent one possible stage in ischemic stroke evolution [9, 10], but DWI-negative/PWI-positive patients are not a homogeneous pre-infarction population. Alternative mechanisms include transient hypoperfusion from spontaneous recanalization or blood pressure lability, robust collateral compensation, post-ictal changes, or a high ischemic threshold [11, 12]. Our preliminary follow-up showed that 35 of 91 DP patients developed new infarction within 3 months, confirming that a subset progress while the majority do not. The lower inflammatory markers and fewer high-risk plaques in DP patients are consistent with a generally less vulnerable vascular state. However, owing to the small number of progressors and incomplete imaging follow-up, we did not systematically analyze individual predictors of progression within the DP subgroup. This highlights the need for multimodal risk stratification and prospective longitudinal studies to clarify the natural history of DWI-negative/PWI-positive patients. In this context, PWI provides essential value for identifying hemodynamic impairment [24]. For patients with ischemic symptoms, performing PWI remains clinically valuable even when DWI shows no positive lesions, as it can reveal cerebral hemodynamic impairment and identify brain tissue at the compensatory edge of perfusion—patients who appear DWI‑negative but may have underlying hemodynamic compromise and potentially increased risk for future ischemic events, though the absolute risk and natural history remain to be defined [25, 26]. Thus, PWI‑defined perfusion deficit may represent an independent risk marker warranting further investigation, particularly in longitudinal studies to clarify which DP patients ultimately progress to infarction. Multidimensional assessment integrating imaging and biomarkers holds significant value for precisely identifying high‑risk individuals at key stages of stroke progression and enabling early targeted intervention.

The sex difference observed is a notable finding. Inflammatory markers (NLR, SIRI, SII) were independently associated with AIS risk only in males, and their inclusion significantly improved model performance solely for male patients, aligning with established sex disparities in immune‑mediated disease risk and pathology [27, 28]. This heterogeneity may stem from immune modulation by sex hormones, inherent differences in atherosclerotic plaque phenotypes between sexes, or distinct immune responses to ischemic injury [29, 30]. Estrogen exerts broad anti‑inflammatory and vasoprotective effects via its receptors [31], while testosterone may have pro‑inflammatory effects. The incidence of atherosclerotic disease in premenopausal women is significantly lower than in men, rapidly increasing after menopause [32]. Thus, the weaker association between inflammatory markers and AIS risk in female patients may partly reflect the attenuating effect of estrogen on inflammation; however, the smaller sample size of women may also limit statistical power, warranting further investigation. Moreover, fundamental differences in plaque morphology and composition exist between sexes: male coronary plaques often have larger necrotic cores and more calcification, while female plaques may be more prone to erosion—differences likely extending to the cerebrovascular domain [27]. The observation that AIS males exhibited both high‑risk plaques and elevated inflammatory levels supports the hypothesis that men may harbor both a heightened systemic inflammatory milieu and more unstable local plaque features, synergistically elevating infarction risk. These findings highlight the potential need for sex‑stratified risk assessment tools. For male ICAS patients, vigilant monitoring and targeted control of systemic inflammation could be a promising avenue for future interventional studies aimed at improving outcomes.

Several limitations warrant consideration. First, the retrospective, single-center design may introduce selection bias and limit generalizability. Moreover, given the cross-sectional nature of this study (blood sampled after the ischemic event), the observed associations do not imply causality. Although we performed a reverse mediation analysis, the non-significant indirect effect does not prove a directional relationship; the findings could still be influenced by unmeasured confounding or post-stroke inflammatory responses. Second, the sample size for prognostic analysis (only 30 patients) increases the risk of overfitting. Although internal validation (cross-validation and bootstrap) demonstrated robust performance (mean cross-validated AUC: 0.919–0.939), external validation in larger cohorts is still needed. Third, unmeasured confounders could not be accounted for, including pre-admission medication history and detailed medical treatment information such as antiplatelet therapy, statin use, and reperfusion treatment, which may have significant impacts on patients’ clinical prognosis and plaque progression. In addition, the two included ischemic stroke phenotypes have inherent heterogeneity in clinical treatment selection, which may further affect the study results, and we were unable to perform standardized stratification and adjustment for this treatment heterogeneity in the multivariate analysis. Additionally, although we supplemented follow‑up data for the DP group by reviewing medical records, this follow‑up was retrospective and incomplete: not all patients underwent systematic imaging at fixed time points, and the small number of progressors limits statistical power. Therefore, our findings on DP progression are exploratory and should be interpreted with caution. Despite these limitations, this study provides novel insight by demonstrating that systemic inflammation is associated with stroke risk in ICAS partly through its link with plaque vulnerability, suggesting that risk stratification may extend beyond stenosis severity to incorporate both systemic inflammatory status and plaque morphology. Future prospective multicenter studies with pre-event biomarker sampling are needed to validate this integrative model and to explore whether targeting inflammation, particularly in high-risk individuals with vulnerable plaques, can improve outcomes.

## Conclusion

In this exploratory study, systemic inflammation measured by accessible hematological indices was associated with HR-VWI-defined high-risk intracranial plaque, and together they helped stratify the risk of AIS and poor prognosis. However, prognostic findings should be interpreted with caution due to the small number of poor outcomes and lack of internal validation. Integrating these biomarkers into a multi-parametric model may help identify patients with hemodynamic impairment at increased risk. The marked sex disparity in the predictive utility of inflammation warrants further investigation into sex-specific ischemic mechanisms.

## Supplementary information

ELECTRONIC SUPPLEMENTARY MATERIAL

## Data Availability

The datasets generated and/or analyzed during the current study are available from the corresponding author on reasonable request, subject to institutional ethical approval.

## Abbreviations

AIS: Acute ischemic stroke
AUC: Area under the curve
CI: Confidence interval
CSVDs: Cerebral small vessel diseases
DP: Delayed perfusion
DWI: Diffusion-weighted imaging
FDR: False discovery rate
HDL: High-density lipoprotein
HR-VWI: High-resolution vessel wall imaging
ICAS: Intracranial atherosclerotic stenosis
IDI: Integrated discrimination improvement
LDL: Low-density lipoprotein
mRS: Modified Rankin scale
MWT: Maximum wall thickness
NIHSS: National Institutes of Health Stroke Scale
NLR: Neutrophil-to-lymphocyte ratio
NRI: Net reclassification improvement
OR: Odds ratio
PLR: Platelet-to-lymphocyte ratio
PWI: Perfusion-weighted imaging
RBG: Random blood glucose
ROC: Receiver operating characteristic
SII: Systemic immune-inflammation index
SIRI: Systemic inflammation response index
TC: Total cholesterol
TG: Triglycerides
TyG: Triglyceride-glucose index
