# Supplementary material for: Systemic inflammatory factors combined with HR-VWI-based plaque vulnerability discriminate ischemic phenotypes and predict prognosis in patients with intracranial atherosclerosis
Source: Insights Imaging. 2026 Sep 5;17:223. doi: 10.1186/s13244-026-02395-1 (PMC13546255; doi:10.1186/s13244-026-02395-1)
Supplement: Supplementary file 1 [file 13244_2026_2395_MOESM1_ESM.pdf]

Systemic Inflammatory Factors Combined with HR-VWI-Based Plaque Vulnerability Discriminates Ischemic Phenotypes and Predicts Prognosis in Patients with Intracranial Atherosclerosis

ELECTRONIC SUPPLEMENTARY MATERIAL

Supplement materials and methods

Study Patients

Following a multidisciplinary consultation, the etiology of stroke was attributed to intracranial artery atherosclerosis based on the following criteria:

Presence of an intracranial plaque upstream of the ischemic territory on HR-VWI, defined as either eccentric thickening of the vascular wall or exhibiting vulnerable features (e.g., plaque enhancement, outward remodeling, or hyperintensity on T1-weighted imaging);

For patients with isolated infarcts confined to a penetrating artery territory, identification of an atherosclerotic plaque in the parent artery on HR-VWI;

Absence of clinical or imaging evidence suggesting aortic arch atherosclerosis;

Absence of clinical or imaging evidence indicating a cardiogenic embolic source for the stroke.

Follow-up of the DP group

For the 91 patients in the DP group (DWI-negative/PWI-positive), we retrospectively reviewed the medical record system to supplement follow-up information. New infarction was defined as the occurrence of a new ischemic stroke event during the 3-month follow-up period. Specifically, if

patients developed acute neurological deficits consistent with stroke, they underwent brain MRI to confirm new infarction. For patients without stroke-related symptoms, follow-up was conducted via outpatient visit or telephone interview at 3 months. The mRS was assessed, and the absence of as stroke symptoms was taken as no new infarction.

Cerebral small vessel diseases assessment

The grading method for cerebral small vessel diseases (CSVDs) is as follows: White matter hyperintensities are scored from 0 to 4 based on the Fazekas scale. Microbleeds, lacunar infarcts, and perivascular spaces are graded by count: absence is scored as 0, 1 to 5 is scored as 1, 5 to 10 is scored as 2, and more than 10 is scored as 3. The total CSVDs score is the sum of the scores from these four indicators.

Collateral vessel grading system:

Collateral vessel grading was assessed on TOF MRA according to the following criteria:

Grade 0: No collateral compensation. No filling of collateral vessels is observed in the ischemic territory.

Grade 1: Poor collateral compensation. Only minimal filling of collateral vessels is present (e.g., filling area < 1/3 of the ischemic territory).

Grade 2: Moderate collateral compensation. Considerable filling of collateral vessels is observed (e.g., filling area approximately 1/3 to 2/3 of the ischemic territory), but the filling speed is slower than that in the contralateral normal territory.

Grade 3: Good collateral compensation. Collateral vessels rapidly and completely fill the entire ischemic territory (⩾2/3), with speed and extent comparable to those in the contralateral normal territory.

Supplement Result

Three-month outcomes of the DP group

Among the 91 DP patients, 35 (38.5%) developed new infarction within 3 months, while 56 (61.5%) did not (**Figure S2**). This preliminary finding highlights the heterogeneity of the DP phenotype: a substantial proportion progress to infarction, but the majority do not.

Internal Validation of the Prognostic Model

To assess the stability and generalizability of the prognostic model (Model 8), we performed three complementary internal validation strategies: (1) 10-fold cross-validation; (2) repeated 10-fold cross-validation with 5 repetitions; and (3) bootstrap resampling with 1000 iterations (models trained on bootstrap samples and tested on the original dataset). The 10-fold cross-validation yielded a mean AUC of 0.931 (95% CI: 0.892-0.970), repeated 10-fold cross-validation with 5 repetitions yielded a mean AUC of 0.919 (SD=0.063), and bootstrap resampling (1000 iterations) yielded a mean AUC of 0.939 (95% CI: 0.864-0.956). Compared to the original apparent AUC of 0.959, the estimated optimism ranged from 0.020 to 0.041 (relative decline of 2.1-4.2%). These findings indicate that the prognostic model exhibits robust performance with only modest overfitting, though external validation remains warranted.

Gender Heterogeneity Analysis of Systemic Immune Inflammatory Response After Cerebral Ischemia

Analysis of the full patient cohort stratified by sex revealed that male patients had a significantly higher prevalence of coronary heart disease, atrial fibrillation, smoking history, and alcohol history compared to females. Biochemically, male patients exhibited significantly lower HDL levels. Monocyte count, TyG index, and the SIRI were significantly elevated in males. No significant differences were observed between sexes in the imaging indicators assessed, as shown in **Table S9**.

Further stratification by stroke subtype indicated that male patients with AIS had higher levels of the NLR, SIRI, and SII compared to female patients with DP, as shown in **Table S10**. This observed disparity translated into subsequent logistic regression analyses, where elevated NLR, SIRI, and SII levels were significantly associated with AIS occurrence in male patients, whereas no such correlation was found in females, as shown in **Table S11**.

ROC curve analysis demonstrated that the incorporation of inflammatory markers (Model 3_male, AUC=0.757, 95%CI: 0.678-0.826, *P*=0.000) significantly improved the predictive performance for AIS occurrence in males compared to the traditional clinical model (Model 1_male, AUC=0.634, 95%CI: 0.549-0.719, *P*=0.004), with Delong test: AUC difference=0.123, *P*=0.019, NRI=43.61%, 95%CI: 13.72-73.5%, *P*=0.004; IDI=0.074, 95%CI: 0.040-0.108, *P*=0.000. Notably, a comprehensive model integrating clinical factors, inflammatory markers, and imaging indicators (Model 5_male and Model 5_female) significantly improved the predictive efficacy for AIS occurrence in both male and female patient groups, as shown in **Table 4.**

Supplementary Tables

Table S1. MRI scanning sequence and parameters

Sequences	Field of view (mm^2)	Repetition time(ms)	Echo time(ms)	Matrix size	Slice thickness (mm)	Bandwidth (hz/pixel)
DWI(b=1000)	220 × 220	4000	93	180 × 180	5.0	1262
TOF-MRA	250 × 204	21	3.42	320 × 223	0.9	185
DSC-PWI	220 × 220	1710	30	128 ×128	4.0	521
IR-SPACE	240 × 210	900	15	384 × 336	0.55	465

Note: DWI=diffusion weight imaging; TOF-MRA=time of flight magnetic resonance angiography; STAGE-MRA=strategically acquired gradient echo magnetic resonance angiography; DSC-PWI=dynamic susceptibility contrast-enhanced perfusion weighted imaging; IR-SPACE=Inversion-recovery prepared sampling perfection with application-optimized contrast using different flip angle evolutions.

Table S2. Inter-bserver and intra-observer consistency of imaging indicators

Imaging indicators	Kappa (Inter-reader reliability)	*P* value	Kappa (Intra-reader reliability)	*P* value
Plaque-RADS	0.954	<0.001	0.929	<0.001
Collateral scale	0.959	<0.001	0.936	<0.001
Fazekas scale	0.951	<0.001	0.942	<0.001
Microbleeds	0.941	<0.001	0.931	<0.001
Lacunar infarcts	0.958	<0.001	0.911	<0.001
Perivascular spaces	0.959	<0.001	0.929	<0.001

Table S3. Demographics and clinical characteristics of patients with different prognosis

Variable	Good prognosis (n=236)	Poor prognosis (n=30)	*P*-value	*P-adjust*
**Age (years)**	58.85±12.52	61.57±11.73	0.595	0.893
**Gender, female, n (%)**	93(39.4)	10(33.3)	0.520	0.832
**Vascular risk factors**				
Hypertension, n (%)	183(77.5)	24(80.0)	0.760	0.893
Diabetes, n (%)	120(50.8)	16(53.3)	0.798	0.893
Hyperlipidemia, n (%)	71(30.1)	11(36.7)	0.462	0.792
Coronary heart disease, n (%)	54(22.9)	9(30.0)	0.388	0.776
Atrial fibrillation, n (%)	24(10.2)	4(13.3)	0.595	0.893
Smoking history, n (%)	137(58.1)	15(50.0)	0.401	0.776
Alcohol history, n (%)	112(47.5)	13(43.3)	0.670	0.893
**NIHSS, median [IQR]**	2[1-4]	6[4-8]	<0.001**	0.024†
**Laboratory data**				
TG, median[IQR]	1.38[0.98-2.13]	1.23[0.94-2.02]	0.725	0.893
TC, median[IQR]	4.02[3.34-4.65]	4.01[3.43-4.45]	0.740	0.893
HDL, median[IQR]	1.01[0.84-1.26]	1.14[0.86-1.41]	0.127	0.331
LDL, median [IQR]	2.41[1.75-3.10]	2.53[1.92-3.08]	0.639	0.893
RBG, median [IQR]	6.00[4.93-7.54]	6.28[5.32-8.76]	0.233	0.508
Neutrophil, median [IQR]	4.32[3.22-5.31]	7.48[4.72-8.76]	<0.001**	0.024†
Lymphocyte, median [IQR]	1.71[1.34-2.19]	1.57[1.06-1.96]	0.124	0.331
Monocyte, median [IQR]	0.43[0.34-0.55]	0.63[0.46-0.77]	<0.001**	0.024†
Platelet, median [IQR]	231.50[193.00-276.00]	261.50[214.00-339.50]	0.045*	0.135
**TyG index**	1.44[1.10-1.88]	1.15[1.11-1.97]	0.946	0.946
**Inflammatory index**				
NLR, median [IQR]	2.32[1.79-3.34]	4.26[2.78-7.67]	<0.001**	0.024†
PLR, median [IQR]	130.51[104.23-179.19]	176.83[129.51-269.64]	0.005*	0.024†
SIRI, median [IQR]	1.05[0.71-1.52]	3.09[1.37-5.37]	<0.001**	0.024†
SII, median [IQR]	536.61[389.43-804.60]	1312.88[535.00-2511.06]	<0.001**	0.024†

Note: TG=Triglycerides; TC=Total Cholesterol; LDL=Low-Density Lipoprotein Cholesterol; HDL=High-Density Lipoprotein Cholesterol; RBD=Random Blood Glucose; NLR=Neutrophil-to-Lymphocyte Ratio;PLR=Platelet-to-Lymphocyte Ratio; SIRI=Systemic Inflammation Response Index; SII=Systemic Immune-Inflammation Index, TyG=Triglyceride glucose index.

*:*P*<0.05, **:*P*<0.001. †: FDR-adjusted *P*-adjust < 0.05 (Benjamini-Hochberg correction).

Table S4. Imaging characteristics of patients with different clinical prognosis

Variable	Good prognosis (n=236)	Poor prognosis (n=30)	*P*-value	*P-adjust*
**Acute ischemic stroke**	152(64.4)	27(90.0)	0.005*	0.025†
**Plaque-RADS**				
High-risk plaque, n (%)	158(66.9)	27(90.0)	0.010*	0.025†
**Collateral scale**			0.804	1.000
0	32(13.6)	6(20.0)		
1	59(25.0)	7(23.3)		
2	65(27.5)	9(30.0)		
3	80(33.9)	8(26.7)		
**CSVDs score, median [IQR]**	6[2-7]	5[3-7]	0.820	1.000

Note:*:*P*<0.05. †: FDR-adjusted P-adjust < 0.05 (Benjamini-Hochberg correction).

Table S5. Demographics and clinical characteristics of patients with different risk plaque

Variable	Total (N=298)	High-risk plaque group (n=213)	Non high-risk plaque group (n=85)	*P*-value	*P-adjust*
**Age (years)**	59.17±12.48	59.38±12.97	58.65±11.22	0.648	0.749
**Gender, female, n (%)**	114(38.3)	81(38.0)	33(38.8)	0.898	0.944
**Vascular risk factors**					
Hypertension, n (%)	232(77.9)	165(77.5)	67(78.8)	0.799	0.887
Diabetes, n (%)	153(51.3)	112(52.6)	42(49.4)	0.736	0.837
Hyperlipidemia, n (%)	91(30.5）	60(28.2)	31(36.5)	0.160	0.255
Coronary heart disease, n (%)	71(23.8)	54(25.4)	17(20.0)	0.327	0.446
Atrial fibrillation, n (%)	30(10.1)	24(11.3)	6(7.1)	0.267	0.386
Smoking history, n (%)	169(56.7)	118(55.4)	51(60.0)	0.469	0.573
Alcohol history, n (%)	139(46.6)	96(45.1)	43(50.6)	0.389	0.505
**NIHSS, median [IQR]**	3[1-4]	3[2-4]	2[1-4]	0.160	0.260
**mRS (0-2), n (%)**	264(88.6)	186(87.3)	80(94.1)	0.689	0.781
**Laboratory data**					
TG, median[IQR]	1.35[0.94-2.13]	1.35[0.96-2.13]	1.35[0.82-2.08]	0.666	0.760
TC, median[IQR]	3.99[3.32-4.71]	3.93[3.33-4.65]	4.06[3.16-4.85]	0.881	0.944
HDL, median[IQR]	1.02[0.85-1.26]	1.01[0.84-1.22]	1.11[0.87-1.33]	0.048*	0.096
LDL, median [IQR]	2.40[1.74-3.09]	2.36[1.75-3.10]	2.54[1.64-3.09]	0.890	0.944
RBG, median [IQR]	6.01[5.00-7.53]	6.10[5.07-8.22]	5.59[4.89-6.82]	0.038*	0.076
Neutrophil, median [IQR]	4.46[3.32-5.69]	4.57[3.57-5.81]	4.09[2.99-5.48]	0.041*	0.082
Lymphocyte, median [IQR]	1.65[1.25-2.13]	1.60[1.22-2.13]	1.75[1.32-2.18]	0.211	0.315
Monocyte, median [IQR]	0.44[0.34-0.56]	0.45[0.36-0.57]	0.42[0.33-0.53]	0.105	0.177
Platelet, median [IQR]	230.50[187.50-276.25]	232.00[186.00-281.00]	226.00[192.00-275.00]	0.680	0.770

**TyG index**	1.44[1.06-1.88]	1.48[1.10-1.89]	1.39[0.91-1.83]	0.123	0.200
**Inflammatory index**					
NLR, median [IQR]	2.57[1.86-3.90]	2.74[1.98-3.99]	2.19[1.67-3.70]	0.020*	0.044†
PLR, median [IQR]	136.76[108.91-190.06]	140.88[108.13-199.06]	130.34[10.960-172.90]	0.300	0.419
SIRI, median [IQR]	1.18[0.78-1.87]	1.25[0.82-1.97]	1.04[0.65-1.55]	0.026*	0.048†
SII, median [IQR]	591.80[414.16-958.05]	611.73[146.02-977.77]	518.62[369.71-803.81]	0.067	0.134

Note: TG=Triglycerides; TC=Total Cholesterol; LDL=Low-Density Lipoprotein Cholesterol; HDL=High-Density Lipoprotein Cholesterol; RBD=Random Blood Glucose; NLR=Neutrophil-to-Lymphocyte Ratio;PLR=Platelet-to-Lymphocyte Ratio; SIRI=Systemic Inflammation Response Index; SII=Systemic Immune-Inflammation Index, TyG=Triglyceride glucose index. *:*P*<0.05. †: FDR-adjusted *P*-adjust < 0.05 (Benjamini-Hochberg correction).

Table S6. Subgroup analysis results of plaque risk

Variable	AIS group (n=207)		Delayed-perfusion group (n=91)		*P*-value
	Non high-risk plaque group (n=42)	High-risk plaque group (n=165)	Non high-risk plaque group (n=43)	High-risk plaque group (n=48)	
**Age (years)**	58.10±10.77	59.59±12.40	59.19±11.75	58.67±14.89	0.863
**Gender, female, n (%)**	16(38.1)	62(37.6)	17(39.5)	19(39.6)	0.992
**Vascular risk factors**					
Hypertension, n (%)	34(81.0)	128(77.6)	33(76.7)	37(77.1)	0.962
Diabetes, n (%)	24(57.1)	89(53.9)	18(41.9)	23(47.9)	0.743
Hyperlipidemia, n (%)	16(38.1)	50(30.3)	15(34.9)	10(20.8)	0.302
Coronary heart disease, n (%)	8(19.0)	42(25.5)	9(20.9)	12(25.0)	0.800
Atrial fibrillation, n (%)	1(2.4)	19(11.5)	5(11.6)	5(10.4)	0.355
Smoking history, n (%)	27(64.3)	93(56.4)	24(55.8)	25(52.1)	0.700
Alcohol history, n (%)	27(64.3)	75(45.5)	16(37.2)	21(43.8)	0.070
**NIHSS, median [IQR]**	3[2-5]	2[1-4]	2[1-4]	2[1-3]	0.004*
**mRS (0-2), n (%)**	39(92.9)	139(84.2)	42(97.7)	47(98.0)	0.163
**Laboratory data**					
TG, median[IQR]	1.47[1.01-2.18]	1.33[0.98-2.22]	1.26[0.79-2.02]	1.42[0.94-1.95]	0.397
TC, median[IQR]	4.16[3.53-5.02]	4.00[3.34-4.72]	3.84[2.72-4.56]	3.69[3.30-4.19]	0.065
HDL, median[IQR]	1.13[0.93-1.35]	1.01[0.74-1.21]	1.09[0.84-1.30]	1.02[0.81-1.29]	0.179
LDL, median [IQR]	2.62[1.98-3.14]	2.41[1.89-3.20]	2.22[1.42-3.07]	2.01[1.63-2.85]	0.069
RBG, median [IQR]	5.80[4.78-7.37]	6.24[5.05-8.41]	5.38[4.93-6.53]	5.93[5.08-6.55]	0.040*
Neutrophil, median [IQR]	4.62[2.97-5.62]	4.73[3.64-6.14]	3.74[2.99-5.32]	4.23[3.43-5.08]	0.026*
Lymphocyte, median [IQR]	1.69[1.27-2.09]	1.60[1.21-2.13]	1.86[1.46-2.21]	1.61[1.35-2.11]	0.315
Monocyte, median [IQR]	0.42[0.34-0.57]	0.46[0.36-0.57]	0.42[0.30-0.49]	0.43[0.33-0.58]	0.274
Platelet, median [IQR]	221.50[187.50-276.25]	234.00[188.00-287.50]	234.00[196.00-276.00]	221.00[183.00-259.50]	0.588

**TyG index**	1.44[1.06-1.90]	1.49[1.10-1.97]	1.28[0.70-1.73]	1.42[1.14-1.70]	0.063
**Inflammatory index**					
NLR, median [IQR]	2.53[1.82-4.38]	2.84[2.10-4.18]	2.06[1.55-3.02]	2.49[1.81-3.48]	0.007*
PLR, median [IQR]	138.09[120.71-188.25]	145.85[110.43-207.26]	120.9[100.50-152.99]	123.42[97.27-162.70]	0.083
SIRI, median [IQR]	1.09[0.72-1.80]	1.30[0.841-2.04]	0.96[0.58-1.48]	1.05[0.72-1.75]	0.028*
SII, median [IQR]	614.20[419.94-791.84]	684.59[432.08-1057.87]	452.96[333.04-846.01]	518.20[352.67-814.18]	0.011*

Note: TG=Triglycerides; TC=Total Cholesterol; LDL=Low-Density Lipoprotein Cholesterol; HDL=High-Density Lipoprotein Cholesterol; RBD=Random Blood Glucose; NLR=Neutrophil-to-Lymphocyte Ratio;PLR=Platelet-to-Lymphocyte Ratio; SIRI=Systemic Inflammation Response Index; SII=Systemic Immune-Inflammation Index, TyG=Triglyceride glucose index. *P*-values are from Kruskal-Wallis test (continuous variables) or chi-square test (categorical variables) for overall comparison across four subgroups. No correction was applied as each variable was tested once *:$P<0.05$.

Table S7. Delong test results of ROC prediction curves

Model Comparison	AUC Difference	Z Statistic	*P* Value
**AIS occurrence**			
Model 2 vs. Model 1	0.071	1.434	0.152
Model 3 vs. Model 1	0.110	2.252	0.024*
Model 4 vs. Model 1	0.223	4.956	<0.001**
Model 5 vs. Model 1	0.230	5.111	<0.001**
**Poor prognosis**			
Model 7 vs. Model 6	0.276	4.325	<0.001**
Model 8 vs. Model 6	0.349	5.469	<0.001**
Model 9 vs. Model 6	0.275	4.274	<0.001**
Model 10 vs. Model 6	0.347	6.273	<0.001**
**Male patients AIS occurrence**			
Model 2_male vs. Model l_male	0.063	1.060	0.289
Model 3_male vs. Model l_male	0.123	2.346	0.019*
Model 4_male vs. Model l_male	0.169	3.048	0.002*
Model 5_male vs. Model l_male	0.180	3.284	0.001*
**Female patients AIS occurrence**			
Model 2_female vs. Model l_female	0.032	0.418	0.676
Model 3_female vs. Model l_female	0.039	0.509	0.611
Model 4_female vs. Model l_female	0.099	1.360	0.174
Model 5_female vs. Model l_female	0.111	1.537	0.124

Note: *:P<0.05, **:P<0.001

Table S8. Effectiveness comparison of logistic regression models

Model Comparison	NRI				IDI			
	Value	SE	*P* value	95%CI	Value	SE	*P* value	95%CI
**All patients AIS occurrence**								
Model 2 vs. Model 1	39.9%	0.119	0.001*	16.6-63.2%	0.046	0.011	<0.001**	0.024-0.068
Model 3 vs. Model 1	42.2%	0.122	0.001*	18.4-66.0%	0.063	0.013	<0.001**	0.036-0.089
Model 4 vs. Model 1	78.8%	0.115	<0.001**	56.2-101.4%	0.198	0.025	<0.001**	0.150-0.247
Model 5 vs. Model 1	86.5%	0.116	<0.001**	60.8-106.1%	0.210	0.026	<0.001**	0.160-0.260
**All patients poor prognosis**								
Model 7 vs. Model 6	121.1%	0.162	<0.001**	88.4-152.8%	0.322	0.059	<0.001**	0.205-0.439
Model 8 vs. Model 6	154.58%	0.118	<0.001**	131.49-177.67%	0.488	0.064	<0.001**	0.362-0.614
Model 9 vs. Model 6	113.62%	0.169	<0.001**	80.59-146.65%	0.325	0.059	<0.001**	0.209-0.440
Model 10 vs. Model 6	155.42%	0.118	<0.001**	132.38-178.47%	0.496	0.064	<0.001**	0.371-0.621
**Male patients AIS occurrence**								
Model 2_male vs. Model l_male	51.36%	0.152	0.001*	21.54-81.18%	0.058	0.015	<0.001**	0.028-0.088
Model 3_male vs. Model l_male	43.61%	0.153	0.004*	13.72-73.50%	0.074	0.125	<0.001**	0.040-0.108
Model 4_male vs. Model l_male	78.73%	0.149	<0.001**	49.50-107.97%	0.220	0.035	<0.001**	0.152-0.288
Model 5_male vs. Model l_male	99.48%	0.140	<0.001**	72.05-126.91%	0.232	0.035	<0.001**	0.164-0.300
**Female patients AIS occurrence**								
Model 2_female vs. Model l_female	-6.84%	0.200	0.732	-45.96-32.28%	0.027	0.015	0.074	-0.003-0.058
Model 3_female vs. Model l_female	17.52%	0.196	0.371	-20.85-55.89%	0.030	0.016	0.056	-0.001-0.061
Model 4_female vs. Model l_female	42.74%	0.197	0.030*	4.18-81.29%	0.105	0.030	<0.001**	0.047-0.163
Model 5_female vs. Model l_female	48.29%	0.195	0.013*	10.13-86.46%	0.114	0.030	<0.001**	0.054-0.174

Note: NRI=Net Reclassification Improvement Index, IDI=Integrated Discrimination Improvement, AIS=Acute Ischemic Stroke, SE=Standard Error, CI=Confidence Interval.

*:P<0.05, **:P<0.001.

Table S9. Demographics and clinical characteristics of patients with different gender

Variable	Male (n=184)	Female (n=114)	*P*-value
**Age (years)**	58.93±12.36	59.55±12.73	0.750
**Vascular risk factors**			
Hypertension, n (%)	145(78.8)	87(76.3)	0.615
Diabetes, n (%)	100(54.3)	54(47.4)	0.387
Hyperlipidemia, n (%)	55(29.9)	36(31.6)	0.759
Coronary heart disease, n (%)	55(29.9)	16(14.0)	0.002*
Atrial fibrillation, n (%)	24(13.0)	6(5.3)	0.030*
Smoking history, n (%)	140(76.1)	29(25.4)	<0.001**
Alcohol history, n (%)	118(64.1)	21(18.4)	<0.001**
**NIHSS, median [IQR]**	3[1-4]	3[1-4]	0.698
**Laboratory data**			
TG, median[IQR]	1.36[0.96-2.08]	1.34[0.92-2.14]	0.888
TC, median[IQR]	3.90[3.11-4.63]	4.11[3.46-4.81]	0.097
HDL, median[IQR]	0.98[0.82-1.20]	1.13[0.94-1.31]	<0.001**
LDL, median [IQR]	2.41[1.68-3.10]	2.38[1.87-3.08]	0.916
RBG, median [IQR]	6.07[4.92-7.72]	6.00[5.18-7.42]	0.585
Neutrophil, median [IQR]	4.70[3.49-5.84]	4.32[2.91-5.31]	0.222
Lymphocyte, median [IQR]	1.63[1.26-2.18]	1.68[1.22-2.08]	0.628
Monocyte, median [IQR]	0.48[0.37-0.60]	0.42[0.31-0.49]	<0.001**
Platelet, median [IQR]	229.00[189.00-281.50]	127.50[186.00-276.00]	0.916
**TyG index**	1.48[1.04-1.88]	1.42[1.08-1.84]	0.600*
**Inflammatory index**			
NLR, median [IQR]	2.62[1.94-4.08]	2.49[1.67-3.80]	0.349
PLR, median [IQR]	137.21[106.22-186.52]	135.73[109.83-203.58]	0.861
SIRI, median [IQR]	1.31[0.82-1.97]	1.05[0.64-1.71]	0.017*
SII, median [IQR]	606.76[434.18-914.63]	536.61[368.81-981.83]	0.342
**Acute ischemic stroke**	129(70.1)	78(68.4)	0.759
**Plaque-RADS**			
High-risk plaque, n (%)	132(71.7)	81(71.1)	0.898
**Collateral scale**			0.250
0	26(14.1)	13(11.4)	
1	54(29.3)	28(24.6)	
2	44(23.9)	37(32.5)	
3	60(32.6)	36(31.6)	
**CSVDs score, median [IQR]**			0.924

Note: TG=Triglycerides; TC=Total Cholesterol; LDL=Low-Density Lipoprotein Cholesterol; HDL=High-Density Lipoprotein Cholesterol; RBD=Random Blood Glucose; NLR=Neutrophil-to-Lymphocyte Ratio; PLR=Platelet-to-Lymphocyte Ratio; SIRI=Systemic Inflammation Response Index; SII=Systemic Immune-Inflammation Index, TyG=Triglyceride glucose index. *:*P*<0.05; ***P*<0.001.

Table S10. Subgroup analysis results of different gender and ischemic phenotypes

Variable	Male (n=184)		Female (n=114)		*P*-value
	DP group (n=55)	AIS group (n=129)	DP group (n=36)	AIS group (n=78)	
**Age (years)**	60.07±12.67	58.45±12.24	57.14±14.51	60.67±11.74	0.210
**Vascular risk factors**					
Hypertension, n (%)	46(83.6）	99(76.7)	24(66.7)	63(80.8)	0.245
Diabetes, n (%)	26(47.3)	73(56.6)	15(41.7)	39(50.0)	0.578
Hyperlipidemia, n (%)	15(27.3)	40(31.0)	10(27.8)	26(33.3)	0.872
Coronary heart disease, n (%)	16(29.1)	39(30.2)	5(13.9)	11(14.1)	0.021
Atrial fibrillation, n (%)	7(12.7)	17(13.2)	3(3.8)	3(3.8)	0.154
Smoking history, n (%)	43(78.2)	97(75.2)	6(16.7)	23(29.5)	<0.001**
Alcohol history, n (%)	30(54.5)	88(68.2)	7(19.4)	14(17.9)	<0.001**
**NIHSS, median [IQR]**	2[1-4]	3[2-4]	2[1-4]	3[2-5]	0.003*
**mRS (0-2), n (%)**	52(94.5)	110(85.3)	36(100.0)	68(87.2)	0.388
**Laboratory data**					
TG, median[IQR]	1.28[0.79-2.08]	1.36[1.00-2.10]	1.34[0.94-1.73]	1.36[0.92-2.26]	0.458
TC, median[IQR]	3.48[2.59-4.31]	4.06[3.33-4.77]	4.11[3.46-4.41]	4.07[3.46-4.99]	0.009*
HDL, median[IQR]	0.96[0.79-1.20]	0.99[0.83-1.22]	1.14[0.92-1.35]	1.13[0.95-1.29]	0.005*
LDL, median [IQR]	1.96[1.41-2.92]	2.63[1.93-3.17]	2.55[1.85-2.97]	2.29[1.86-3.40]	0.010*
RBG, median [IQR]	5.78[4.88-6.64]	6.17[4.96-8.52]	5.51[5.12-6.13]	6.22[5.00-7.80]	0.088
Neutrophil, median [IQR]	4.15[3.21-5.32]	4.90[3.70-6.15]	3.92[2.71-5.08]	4.44[2.99-5.57]	0.006*
Lymphocyte, median [IQR]	1.65[1.39-2.30]	1.60[1.21-2.15]	1.86[1.45-2.15]	1.62[1.21-2.07]	0.444
Monocyte, median [IQR]	0.47[0.34-0.56]	0.49[0.37-0.60]	0.41[0.30-0.47]	0.42[0.33-0.50]	0.001*
Platelet, median [IQR]	221.00[186.00-272.00]	230.00[191.50-286.00]	236.50[188.50-269.00]	251.50[184.25-285.50]	0.832
**TyG index**	1.35[0.77-1.85]	1.58[1.12-1.91]	1.36[1.11-1.65]	1.44[1.07-2.17]	0.098
**Inflammatory index**					
NLR, median [IQR]	2.23[1.79-3.18]	2.74[2.01-4.48]	2.06[1.46-3.66]	2.84[2.03-3.82]	0.013*

PLR, median [IQR]	121.72[96.90-156.69]	146.82[109.94-195.11]	117.20[99.03-180.72]	140.62[112.82-226.57]	0.085
SIRI, median [IQR]	1.06[0.75-1.55]	1.37[0.90-2.28]	0.95[0.55-1.60]	1.09[0.70-1.73]	0.006*
SII, median [IQR]	514.19[348.37-817.28]	700.41[448.72-996.00]	464.55[300.56-925.72]	617.76[408.66-990.57]	0.012*

Note: TC=Total Cholesterol; LDL=Low-Density Lipoprotein Cholesterol; NLR=Neutrophil-to-Lymphocyte Ratio;PLR=Platelet-to-Lymphocyte Ratio; SIRI=Systemic Inflammation Response Index; SII=Systemic Immune-Inflammation Index; CSVDs: Cerebral small vessel diseases.

Multivariate logistic regression model has been adjusted for gender, age and vascular risk factors (hypertension, diabetes, hyperlipidemia, coronary heart disease, atrial fibrillation, smoking history and drinking history).

P-values are from Kruskal-Wallis test (continuous variables) or chi-square test (categorical variables) for overall comparison across four subgroups. No correction was applied as each variable was tested once. *:$P<0.05$, **:$P<0.001$

Table S11. Univariate and multivariate logistic analysis for acute ischemic stroke occurrence in male and female patients

Variable	Univariable analysis				Multivariable analysis		
	OR value	95%CI	*P* value	*P-adjust*	OR value	95%CI	*P* value
				**Male Patients**			
**NIHSS**	1.320	1.101-1.582	0.003*	0.003†	1.334	1.099-1.619	0.004*
**Inflammatory index**							
NLR	1.243	1.020-1.514	0.031*	0.019†	1.250	1.018-1.534	0.033*
PLR	1.003	0.999-1.008	0.172	0.044†	1.004	0.999-1.009	0.106
SIRI	1.436	1.026-2.009	0.035*	0.025†	1.498	1.055-2.127	0.024*
SII	1.001	1.000-1.001	0.066	0.031†	1.001	1.000-1.001	0.045*
**High-risk plaque**	3.552	1.796-7.025	<0.001**	<0.001†	4.331	2.059-9.112	<0.001**
**Collateral scale**	0.492	0.353-0.688	0.492	0.050	0.431	0.300-0.619	<0.001**
**CSVDs score**	1.097	0.990-1.216	0.078	0.038†	1.186	1.034-1.360	0.015*
				**Female Patients**			
**NIHSS**	1.259	1.018-1.557	0.033*	0.025†	1.249	0.991-1.573	0.059
**Inflammatory index**							
NLR	1.042	0.917-1.184	0.526	0.044†	1.022	0.896-1.165	0.748
PLR	1.000	0.997-1.004	0.807	0.050	1.000	0.996-1.004	0.912
SIRI	1.152	0.849-1.564	0.364	0.031†	1.118	0.837-1.495	0.450
SII	1.000	1.000-1.001	0.477	0.038†	1.000	0.999-1.001	0.624
**High-risk plaque**	3.467	1.475-8.149	0.004*	0.004†	3.669	1.458-9.235	0.006*
**Collateral scale**	0.511	0.323-0.808	0.004*	0.004†	0.513	0.318-0.829	0.006*
**CSVDs score**	1.155	1.013-1.317	0.032*	0.019†	1.160	0.994-1.353	0.059

Note: TC=Total Cholesterol; LDL=Low-Density Lipoprotein Cholesterol; NLR=Neutrophil-to-Lymphocyte Ratio;PLR=Platelet-to-Lymphocyte Ratio; SIRI=Systemic Inflammation Response Index; SII=Systemic Immune-Inflammation Index; CSVDs: Cerebral small vessel diseases.

Multivariate logistic regression model has been adjusted for age and vascular risk factors (hypertension, diabetes, hyperlipidemia, coronary heart disease, atrial fibrillation, smoking history and drinking history).

*:*P*<0.05, **:*P*<0.001. †: FDR-adjusted *P*-adjust < 0.05 (Benjamini-Hochberg correction).

Supplementary Figures

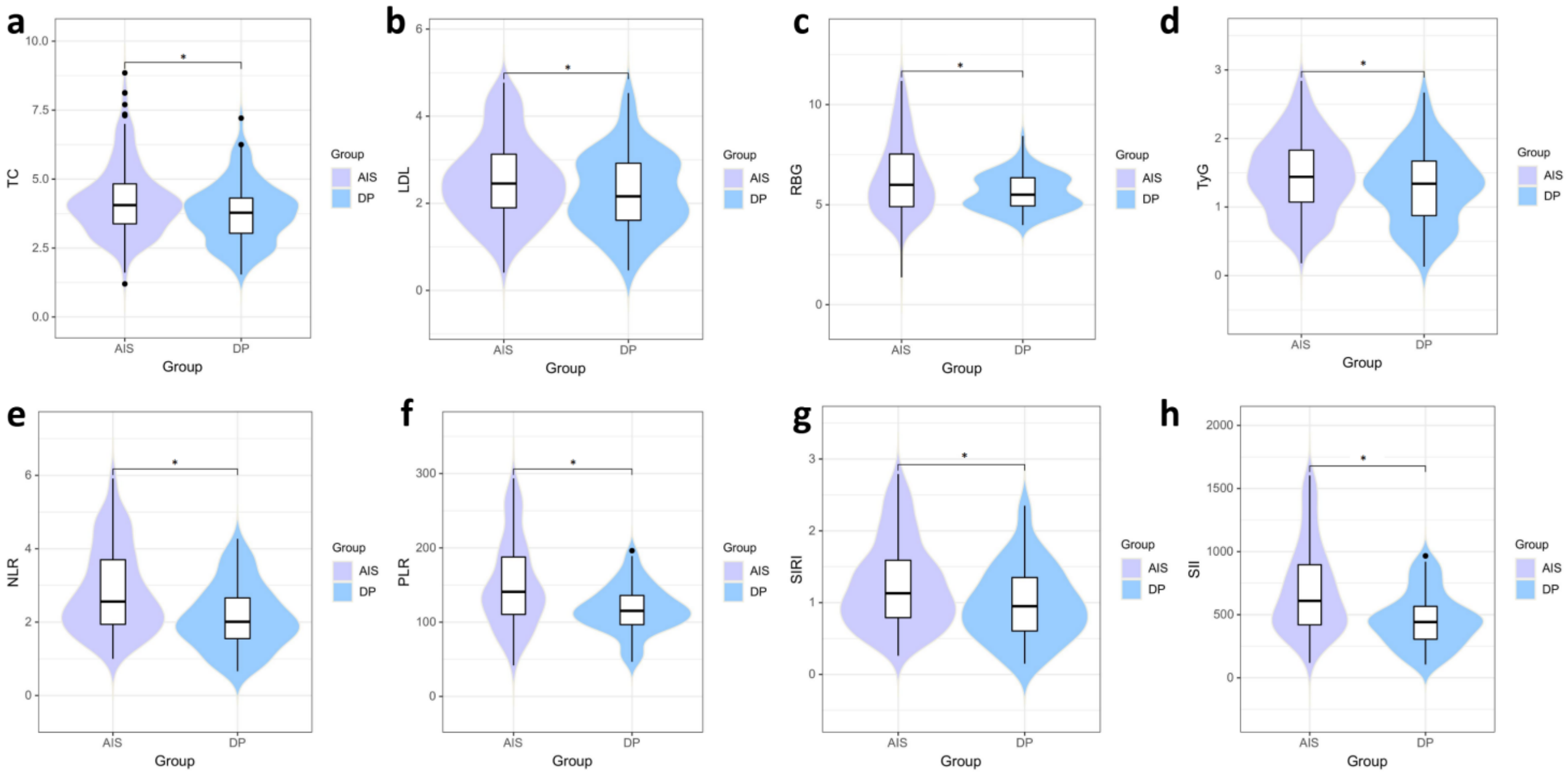

Figure S1. Violin plots showing differences in metabolic and inflammatory indicators between patients with acute ischemic stroke and delayed perfusion.

Note: TC=Total Cholesterol; LDL=Low-Density Lipoprotein Cholesterol; RBG=Random Blood Glucose; NLR=Neutrophil-to-Lymphocyte Ratio;PLR=Platelet-to-Lymphocyte Ratio; SIRI=Systemic Inflammation Response Index; SII=Systemic Immune-Inflammation Index.
*: P<0.05.

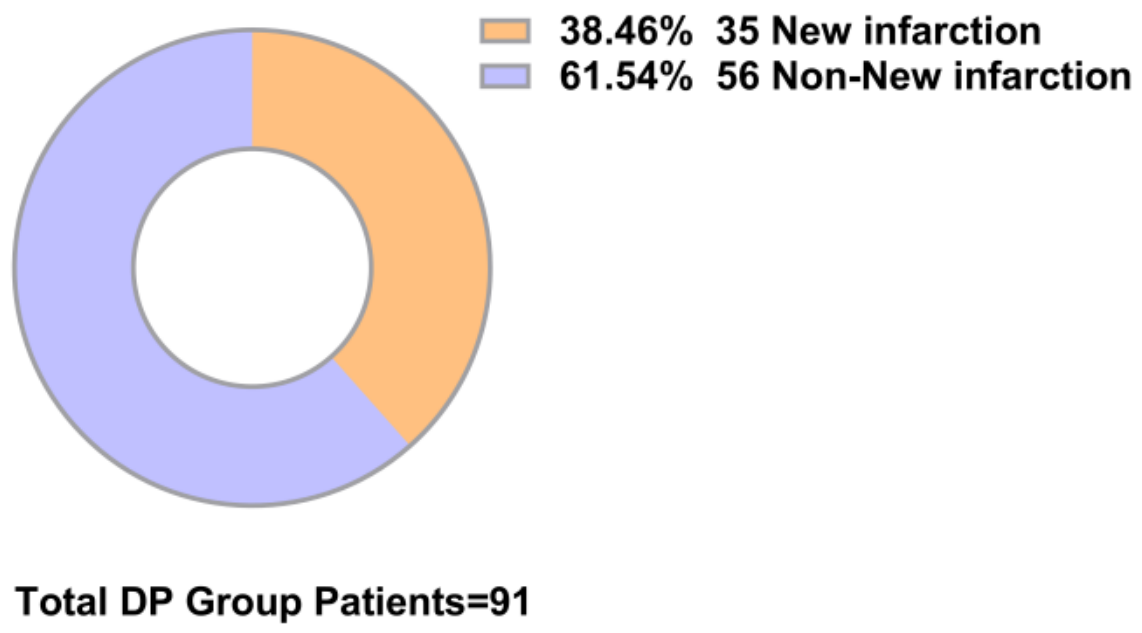

Figure S2. Three-month outcomes of the DP (DWI-negative/PWI-positive) group.

A total of 91 patients were followed; 35(38.5%) developed new infarction, whereas 56(61.5%) did not.

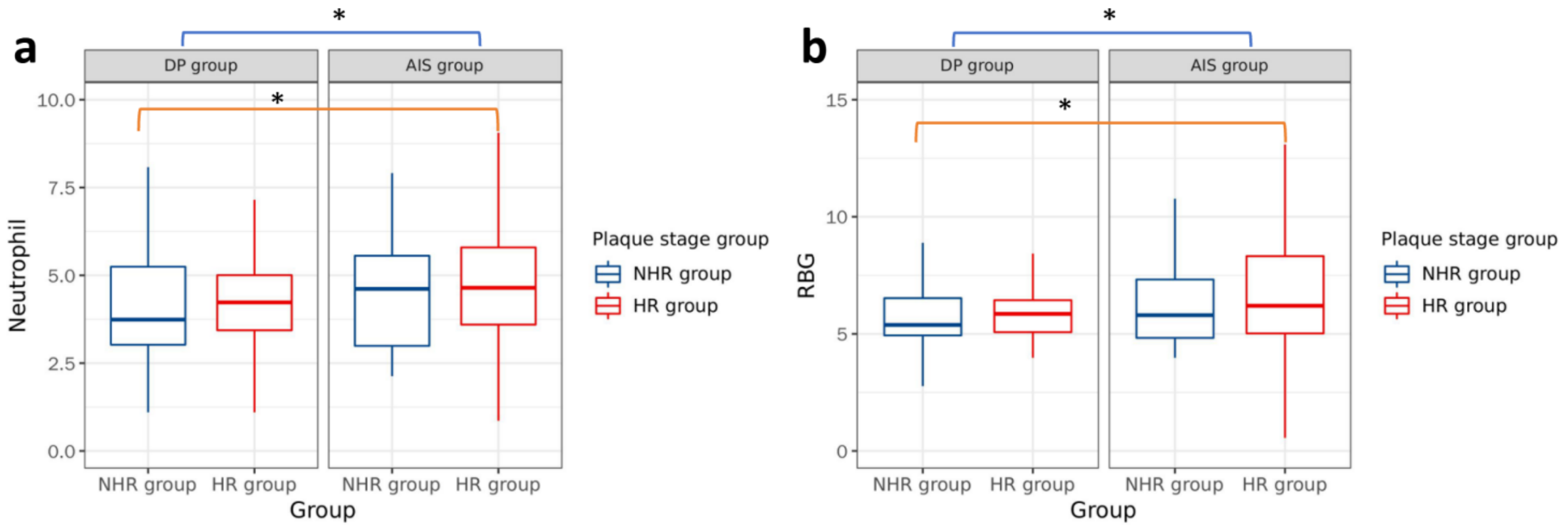

Figure S3. Box chart of supplementary results based on subgroup analysis of plaque vulnerability.

Figure a shows that the neutrophil count of AIS patients with high-risk (HR) plaque is significantly higher than that of DP patients with non-high-risk (NHR) plaque. Figure b shows that the random blood glucose (RBG) of AIS patients with HR plaque is significantly higher than that of DP patients with NHR plaque.

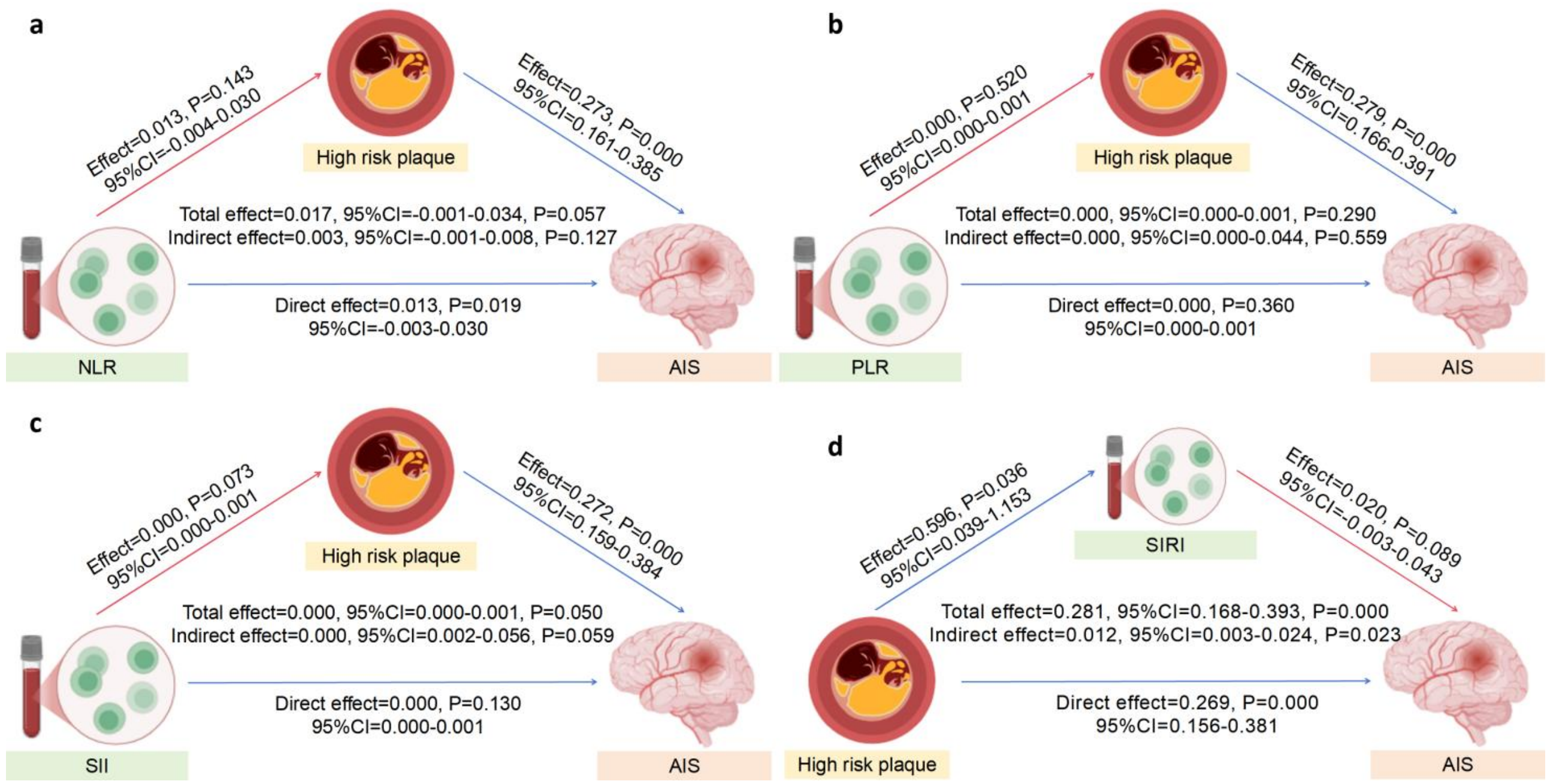

Figure S4. Mediation effect analysis results

High risk plaque cannot mediate the effect of NLR, PLR and SII on AIS in DP patients. SIRI cannot mediate the effect of high risk plaque on AIS in DP patients.

SIRI=Systemic Inflammation Response Index; NLR=Neutrophil-to-Lymphocyte Ratio;PLR=Platelet-to-Lymphocyte Ratio; SII=Systemic Immune-Inflammation Index.

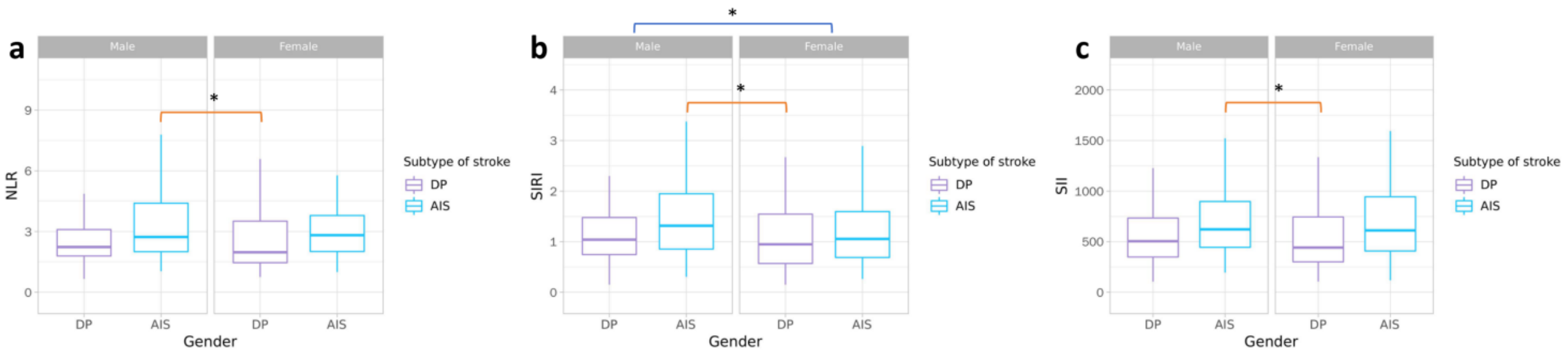

Figure S5. Inter group comparison box chart results of subgroups by gender.

NLR=Neutrophil-to-Lymphocyte Ratio; SIRI=Systemic Inflammation Response Index; SII=Systemic Immune-Inflammation Index; DP: delayed perfusion; AIS: acute ischemic stroke.

*:*P*<0.05.
